# Supplementary material for: Task specificity in mouse parietal cortex
Source: Neuron. 2022 Sep 21;110(18):2961–2969.e5. doi: 10.1016/j.neuron.2022.07.017 (PMC9616730; doi:10.1016/j.neuron.2022.07.017)
Supplement: Document S1. Figures S1–S7 [file mmc1.pdf]

Neuron, Volume *110*

Supplemental information

Task specificity in mouse parietal cortex

Julie J. Lee, Michael Krumin, Kenneth D. Harris, and Matteo Carandini

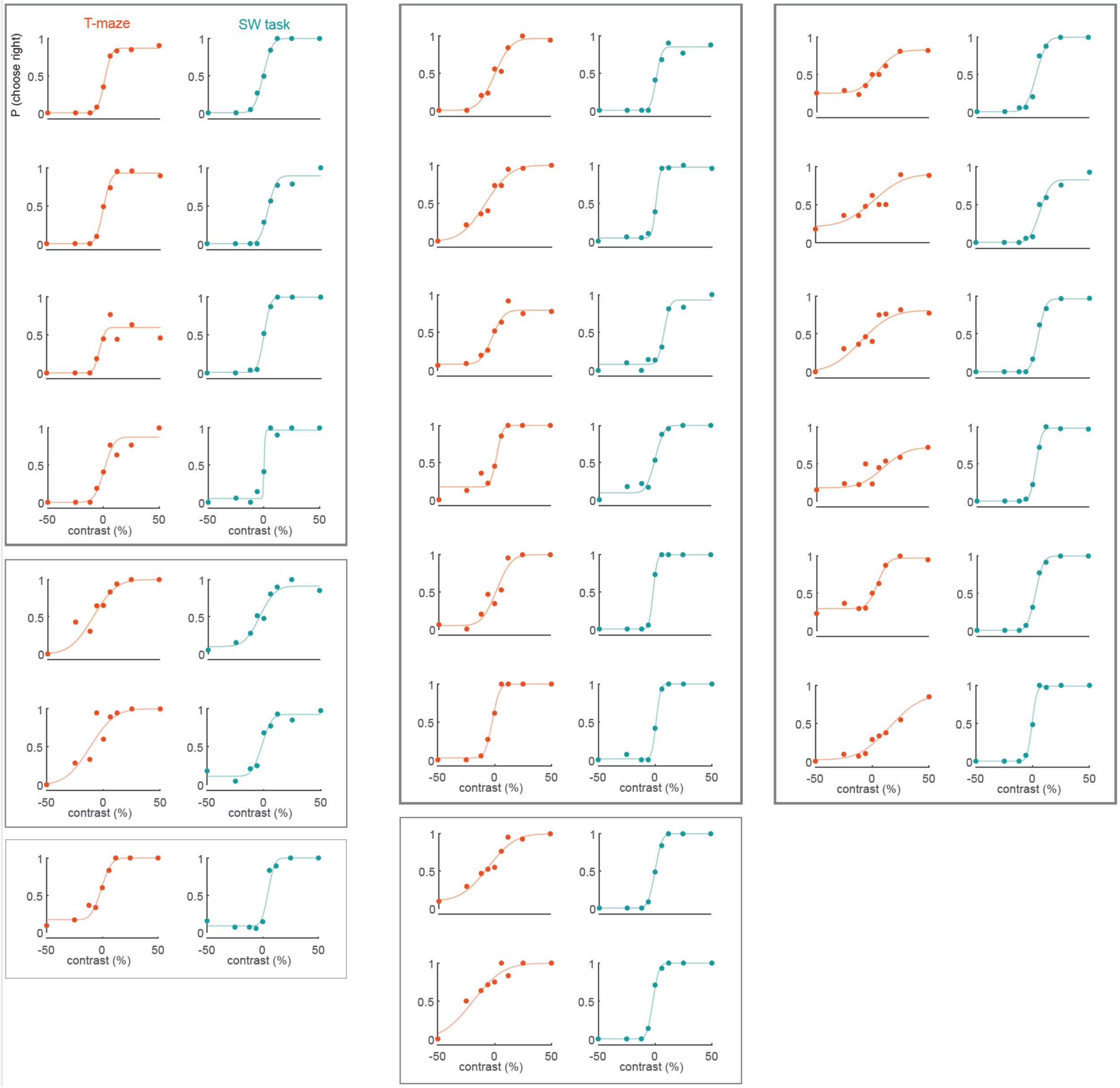

Supplementary Figure S1. Psychometric data for each task, session, and mouse. Related to Figure 1.
For each of six trained mice (boxes), we measured neural activity in one or more sessions (rows). Each session included two tasks performed consecutively: T-maze (*orange*, left) and Steering Wheel task (*cyan*, right). Tasks were not necessarily presented in the order shown.

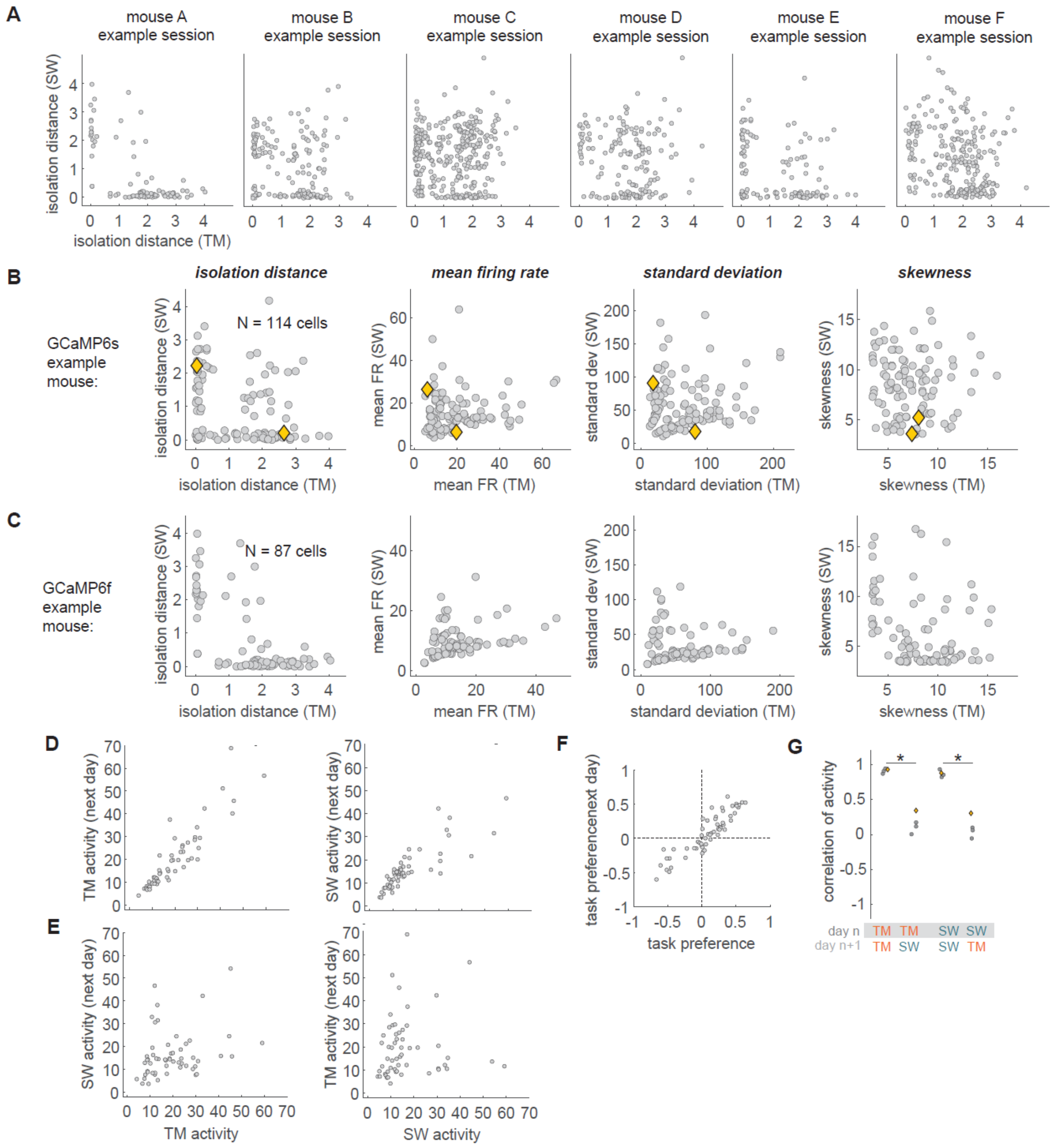

Supplementary Figure S2. Further examples and alternative measures of activity. Related to Figure 1.
(A) Further example sessions (one per mouse) showing activity (isolation distance) across tasks (as in Figure 1E).
(B) Comparison of activity in the T-maze (TM, abscissa) vs. steering wheel task (SW, ordinate) for the example session in Fig. 1E (in a mouse expressing GCaMP6s, mouse E), using different measures for calculating activity: isolation distance, mean, standard deviation and skewness. *Diamonds* indicate the same neurons in all plots.
(C) Same as B, for an example session from a GCaMP6f mouse (mouse A).
(D-G) Reproduction of analyses from Figs 2B-E using mean firing rate as the measure of activity instead of isolation distance. Mean firing rate was more similar across than within tasks.

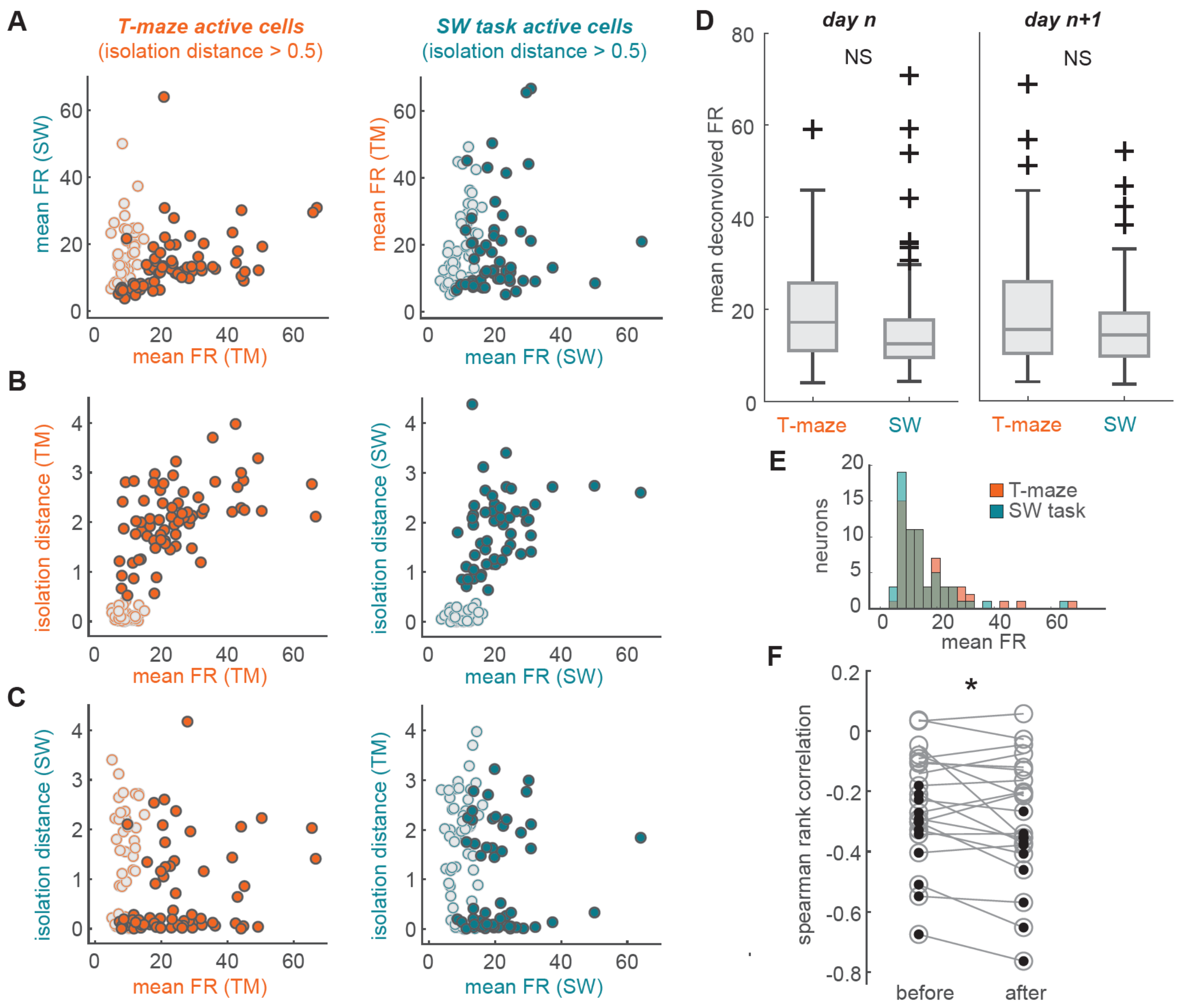

Supplementary Figure S3. Effects of mean firing rate on isolation distance and correlations. Related to Figure 1.

(A) Comparison of mean firing rate across tasks in an example session for cells with some activity (isolation distance > 0.5, *filled circles*) in the T-maze task (*left)* or the steering-wheel task (*right*).

(B) Same format, comparing mean firing rate and isolation distance within the same task. There is a positive relationship between the two measures.

(C) Same format, comparing mean firing rate and isolation distance across tasks. A negative relationship means high firing rate in one task predicts low activity in the other task (i.e., task selectivity); a positive relationship means high firing rate in one task predicts high activity in the other task (i.e., task generality). Here, the T-maze (*bottom left*) had a slight significant correlation, r = 0.25, p = 0.04, and the steering wheel task (*bottom right*) did not have a significant correlation, p = 0.65. However, across sessions, there was a small but significant positive relationship using a linear mixed-effects model: other task's isolation distance ~ mean FR + (mean FR | session), p = 0.02. That is, neurons with higher firing rate were slightly more likely to be active in both tasks.

(D) Mean firing rate (FR) across tasks, for the example session in Fig. 2. Here, mean FRs in the T-maze and SW task were not significantly different (rank-sum test). However, in other sessions (13/21), firing rate was higher in the T-maze. Indeed, task was a significant predictor of mean FR (p < 0.01, linear mixed-effect model with session as a random effect, FR ~ task + (task | session)).

(E) To test for the effect of firing rate, we subsampled neurons so that their mean firing distributions for each task (orange and blue histograms) were statistically indistinguishable (p ⩾ 0.05 in a rank-sum test). This histogram shows results for an example session.

(F) Subsampling to equate firing rate distributions often made the Spearman rank correlation of isolation distance more negative across tasks (filled circles denote sessions where p < 0.05 comparing before and after subsampling). This tendency was significant across sessions (p = 0.04, one-tailed sign-rank test). Therefore, differences in mean firing rate across tasks do not account for our findings, as similar firing rates produce the same results.

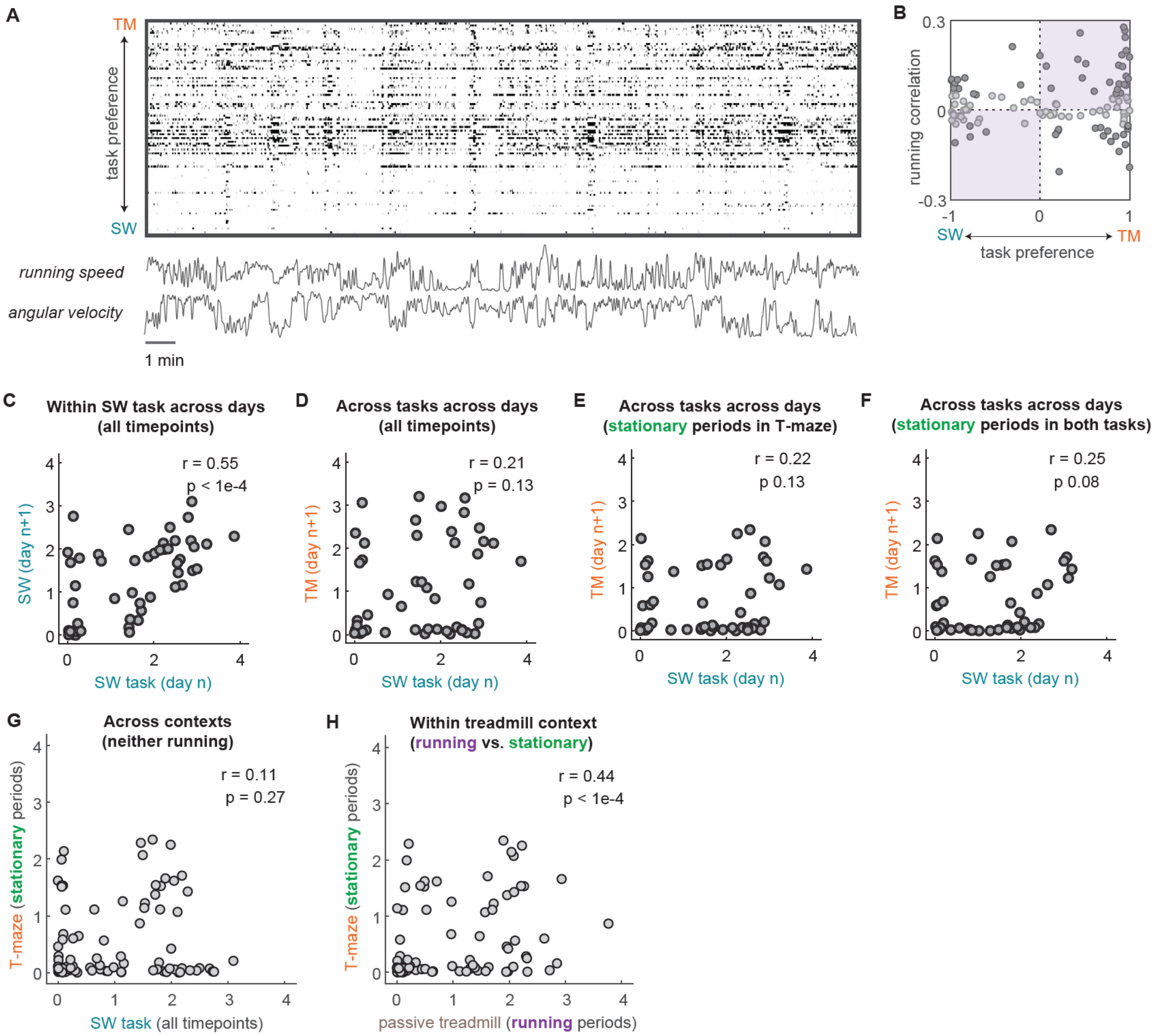

Supplementary Figure S4. Running does not explain task specificity. Related to Figure 3.

(A) Firing rate of neurons, sorted by task selectivity, in an example session during the passive treadmill condition. Below: running speed and angular velocity on the ball.

(B) Running modulation does not explain task preference. Positive vs. negative values on the y-axis indicate preference for T-maze vs. steering wheel (SW) task. *Filled circles*: neurons with significant running modulation (permutation test). *Purple overlay*: hypothetical distributions of cells, if T-maze neurons were just those modulated by running, and SW task neurons were just those suppressed by running. In this session, the Spearman rank correlation was not significant, using all neurons, r = 0.04, p = 0.67, or just significantly running-modulated neurons (filled circles), r = 0.06, p = 0.65.

(C) Comparing steering-wheel (SW) task activity across two days; activity is highly correlated (r = 0.55, p < 1e-4).

(D) Same example days, comparing across tasks; activity is not significantly correlated (r = 0.21, p = 0.13).

(E) Same as D, measuring T-maze activity only during stationary periods (running speed < 1.2 cm/s); activity is not significantly correlated (r = 0.22, p = 0.13).

(F) Same as D, using only stationary periods in both tasks; activity is not significantly correlated (r = 0.25, p = 0.08).

(G) Another session where activity in the T-maze task during stationarity is not correlated with activity in the steering-wheel task (r = 0.11, p = 0.27). This mirrors the results of activity from Fig 1E. The same result was observed in another session (not shown, r = 0.04 p = 0.73). This result holds even when we additionally excluded periods of steering wheel movements, i.e., forelimb stationarity (this session: r = 0.18, p = 0.08; another: r = -0.01, p = 0.95). In summary, activity was not significantly correlated across contexts, even within the same movement condition (stationarity).

(H) Same session as in (G). Activity in the T-maze task during stationarity is correlated with the passive treadmill condition during running (r = 0.44, p < 1e-4). The same pattern was seen in another session (not shown, r = 0.37, p < 0.01). This extends the results of correlated activity between task and passive conditions within the T-maze context (Fig 2B), and shows that this correlation holds even across movement conditions (running vs stationary).

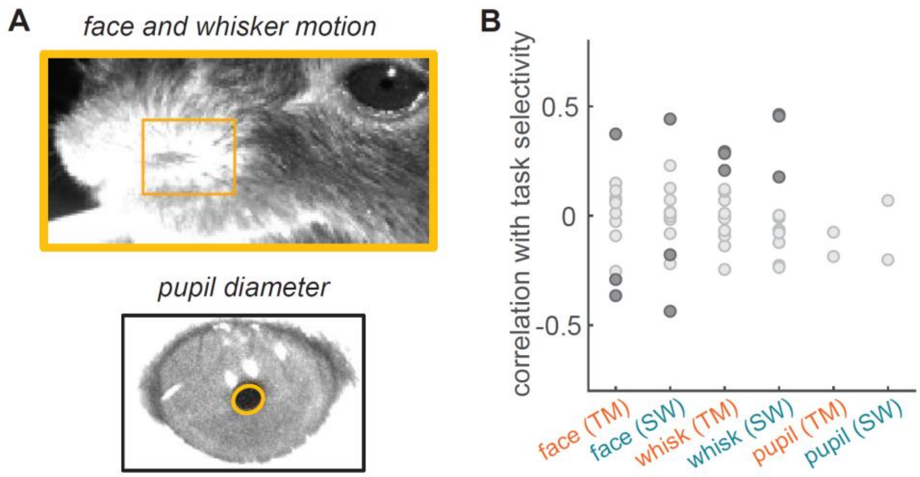

Supplementary Figure S5. Facial movements and pupil diameter do not explain task specificity. Related to Figure 3.
(A) Video frame from sessions with high-resolution video of face (*top*) or eye (*bottom*). Face motion was extracted from the whole face image (large yellow rectangle), whisker motion from a smaller region (smaller rectangle), and pupil diameter from a fitted ellipse (yellow contour). Facial movements, whisker motion and pupil diameter were estimated separately in each task and session (Methods).
(B) Summary of correlations of movement modulation with task selectivity, as in Supplementary Fig. S4B, although estimated within each task condition. Each point is a session. Filled circles are significant correlations ($p < 0.05$, 3/13 sessions for facial movements and whisking, 0/2 sessions for pupil diameter).

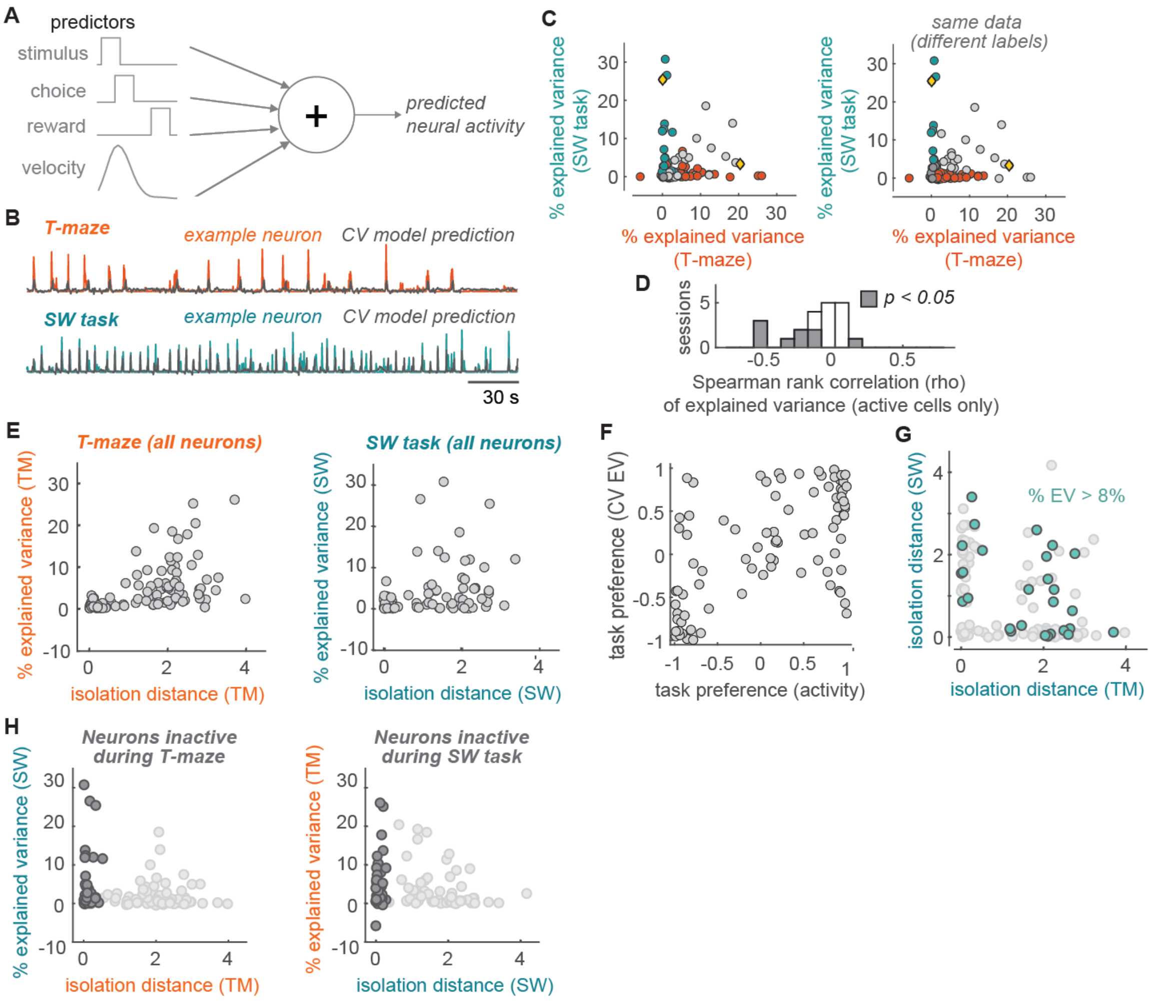

Supplementary Figure S6. Encoding of task variables is task specific. Related to Figure 4.
(A) Schematic of an encoding model based on behavioral predictors: stimulus (left vs right, or zero contrast), choice (left vs right), reward, and velocity of the respective apparatus. The model was fit separately to each task.
(B) Magnified traces of two neurons: one that responded in the T-maze task (*top*) and one that responded in the Steering Wheel task (*bottom*), showing deconvolved calcium activity (*color*) and model predictions (*gray*).
(C) Cross-validated explained variance (%) by each encoding model for the two tasks, in the example session of Fig. 1E. Example neurons in (B) are indicated by diamonds. Spearman rank correlation across tasks: r = 0.08, p = 0.38. We fitted a two-dimensional Gaussian mixture model to this data. The model returns cluster labels by determining the probability of cluster assignment according to a prespecified number of clusters. This method is the same as used to label the clusters in existing Figure 1E. *Left:* Neurons (dots) are colored according to Fig. 1E. *Right:* neurons are fitted according to % explained variance.
(D) Summary over sessions showing Spearman rank correlation of cross-validated explained variance across tasks, for neurons active in either or both tasks (isolation distance > 0.5). *Filled* bars indicate significance (9/21 sessions).
(E) Relationship between activity (isolation distance) and encoding of task variables (% cross-validated variance explained) for the T-maze (*left*) and the SW task (*right*), for all neurons in the example session from Figs 2 and 5.
(F) Same data as in (A) comparing task preference measured by activity vs task preference measured by cross-validated variance explained by the encoding models. The two measures were well-correlated, r = 0.62, p < 0.001.
(G) Same data as in (A) comparing isolation distance across tasks for the subset of neurons encoding task-relevant information in either task (*cyan*: >8% cross-validated explained variance in the T-maze, steering-wheel task, or both). Spearman rank correlation, r = -0.48, p = 0.003. Across all sessions, 15/21 sessions had significant negative correlations (p<0.05) and 6/21 were not significantly correlated; median r = -0.47.
(H) Same data as in (A), comparing activity and encoding across tasks. Filled circles indicate neurons inactive in one task (isolation distance < 0.5; silent neurons were excluded). These neurons tend to encode task-relevant activity in the other task. Explained variance by task-related events was significantly (p < 0.05) greater than 0 in 12/21 sessions for task encoding in the SW task for neurons inactive in the T-maze (e.g., *left*) and for task encoding in the T-maze in 20/21 sessions for neurons inactive in the SW task (e.g., *right*). Significance of task encoding was assessed according to a permutation test where the median cross-validated explained variance (of inactive cells in the other task) was compared to permutations where the labels of inactive vs active cells were shuffled randomly 1000 times and the median explained variance of these pseudo-inactive populations was computed. The explained variance was considered significant if the actual median was greater than the 95th percentile (alpha level = 0.05) of the permutations.

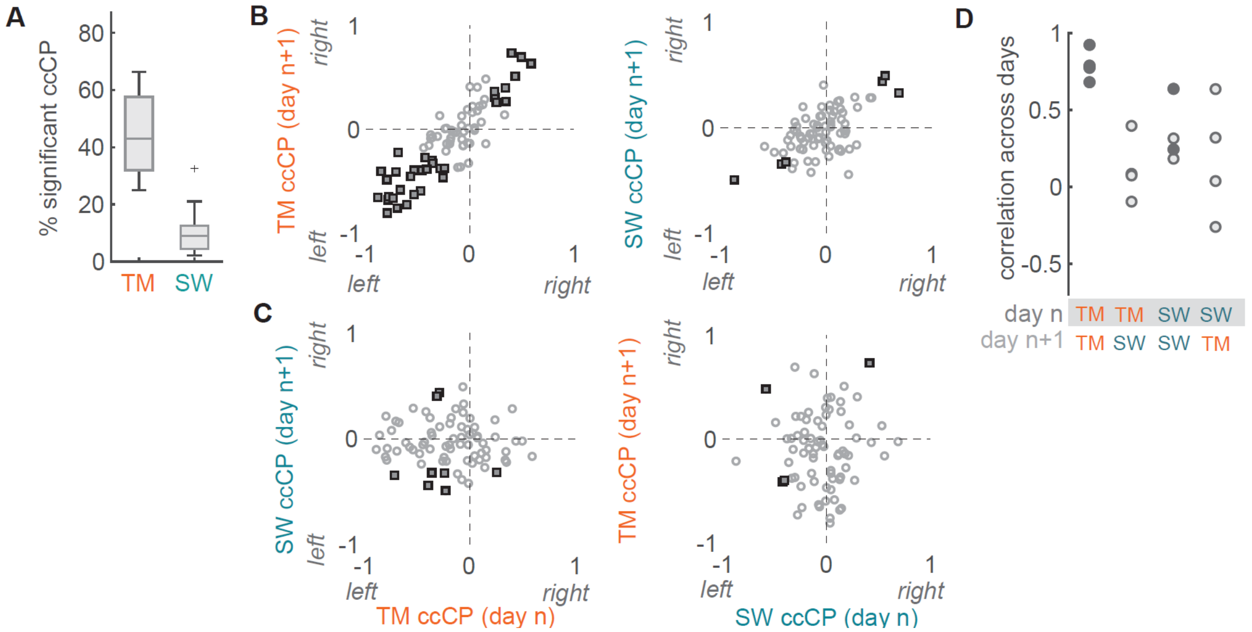

Supplementary Figure S7. Preference for ongoing choice is not shared across tasks. Related to Figure 4.

(A) Proportion of neurons with significant choice selectivity (ccCP) over 21 sessions from 6 mice, for neurons with at least a little activity (isolation distance > 0.3) in each task (both task-selective and task-general neurons). Outliers are denoted by a *plus* symbol. Across sessions, there was no significant difference between the number of choice-selective neurons in the task-selective and task-general populations (isolation distance > 0.5 in only one vs. both tasks), for either the T-maze ($p = 0.84$, paired sign rank test) or SW task ($p = 0.67$). For the T-maze, median number of choice-selective neurons in the task-selective population: 28; in the task-general population: 9.5. For the SW task, median number of choice-selective neurons in the task-selective population: 4.0; in the task-general population: 4.5.

(B-C) Choice selectivity (ccCP) across days within tasks (B) and across tasks (C) for neurons showing some activity in both tasks (isolation distance > 0.3 in both tasks). Negative values denote preferences for left choices, positive for right choices. *Filled squares* denote neurons that were significantly selective for choice in either day ($p<0.05$). Out of the larger recorded population, the proportion of neurons active in both tasks plotted in (C) was 23.4% for one cross-task comparison (*left*) and 26.0% for the other (*right*).

(D) Correlation of ccCP across days for different combinations of tasks, for n = 3 mice, four pairs of days). Neurons were selected as in C-F (isolation distance > 0.3 in both tasks. Spearman rank correlation (f*illed circles*) was only significant within tasks (across days), not across tasks.
