# Supplementary material for: Task specificity in mouse parietal cortex
Source: Neuron. 2022 Sep 21;110(18):2961–2969.e5. doi: 10.1016/j.neuron.2022.07.017 (PMC9616730; doi:10.1016/j.neuron.2022.07.017)
Supplement: Document S2. Article plus supplemental information [file mmc2.pdf]

Neuron

Report

Task specificity in mouse parietal cortex

Highlights

- We trained the same mice to perform two different visual decision tasks
- Neurons in parietal cortex active in one task were typically inactive in the other
- This specificity depended on task apparatus, even in passive conditions
- A third, hybrid task confirmed that specificity depends on physical context

Authors

Julie J. Lee, Michael Krumin, Kenneth D. Harris, Matteo Carandini

Correspondence

juliejlee@nyu.edu

In brief

Lee et al. trained the same mice to perform two or even three visual decision tasks involving different apparatuses. They discovered that neurons in parietal cortex exhibit strong task specificity: different parietal neurons are active in different physical contexts.

Lee et al., 2022, Neuron *110*, 2961–2969
September 21, 2022 © 2022 The Author(s). Published by Elsevier Inc.
https://doi.org/10.1016/j.neuron.2022.07.017

CellPress

Neuron

CellPress OPEN ACCESS

Report

Task specificity in mouse parietal cortex

Julie J. Lee,[1,3,4,*] Michael Krumin,[1] Kenneth D. Harris,[2] and Matteo Carandini[1]
[1]UCL Institute of Ophthalmology, University College London, Gower Street, London WC1E 6AE, UK
[2]UCL Queen Square Institute of Neurology, University College London, Gower Street, London WC1E 6AE, UK
[3]Present address: NYU Department of Psychology, 6 Washington Place, New York, NY 10003, USA
[4]Lead contact
*Correspondence: juliejlee@nyu.edu
https://doi.org/10.1016/j.neuron.2022.07.017

SUMMARY

Parietal cortex is implicated in a variety of behavioral processes, but it is unknown whether and how its individual neurons participate in multiple tasks. We trained head-fixed mice to perform two visual decision tasks involving a steering wheel or a virtual T-maze and recorded from the same parietal neurons during these two tasks. Neurons that were active during the T-maze task were typically inactive during the steering-wheel task and vice versa. Recording from the same neurons in the same apparatus without task stimuli yielded the same specificity as in the task, suggesting that task specificity depends on physical context. To confirm this, we trained some mice in a third task combining the steering wheel context with the visual environment of the T-maze. This hybrid task engaged the same neurons as those engaged in the steering-wheel task. Thus, participation by neurons in mouse parietal cortex is task specific, and this specificity is determined by physical context.

INTRODUCTION

The brain must meet a vast variety of potential behavioral demands while relying on a finite number of neurons. It might thus flexibly re-engage the same neurons in multiple behaviors. A region where one might expect to find neurons involved in multiple behavioral tasks is the parietal cortex, where neurons have been implicated in many aspects of vision, decision-making, action, and navigation. In particular, parietal neurons have been proposed to encode aspects of motor planning, evidence accumulation, choice sequences, spatial position and heading, movement motifs, movement sequences, and body posture (e.g., Britten et al., 1996; Chen et al., 1994; Gnadt and Andersen, 1988; Hanks et al., 2015; Harvey et al., 2012; Krumin et al., 2018; Mimica et al., 2018; Nitz, 2006, 2012; Pinto et al., 2019; Shadlen and Newsome, 2001; Snyder et al., 1997; Whitlock et al., 2012; Wilber et al., 2014). Individual parietal neurons can encode multiple task variables (Meister et al., 2013; Park et al., 2014; Raposo et al., 2014; Zhang et al., 2017). This "mixed selectivity," however, is typically defined within a single behavioral task; it does not predict how the same neurons would be engaged across multiple tasks.

Recordings of the same parietal neurons across tasks are difficult and thus rarely performed. Studies in rodents varied sensory demands and found that parietal neurons have similar responses when a choice was based on visual versus auditory stimuli (Raposo et al., 2014) or visual versus tactile stimuli (Nikbakht et al., 2018). Studies in primates varied motor demands: some found that neurons in different parietal areas show selective activity for eye versus arm movements (Snyder et al., 1997), whereas others found that neurons are engaged by both movements (Mohan et al., 2021). It is not clear how to relate these studies, because they manipulated different variables (sensory versus motor) in different species (primates versus rodents). Indeed, while parietal cortex is defined similarly in these species, i.e., by proximity to visual and somatosensory areas and connectivity to thalamus (Hovde et al., 2019; Olsen and Witter, 2016; Whitlock, 2017), macaque parietal areas have no clear homolog in the rodent.

Here, we recorded from many parietal neurons while mice performed two visual decision tasks in different task contexts. Both tasks required a two-alternative forced choice to indicate the presence of a grating stimulus, but the tasks involved different visual stimuli, different motor outputs, and different apparatus (Burgess et al., 2017; International Brain Laboratory et al., 2021; Krumin et al., 2018). The two tasks activated largely distinct but spatially intermixed subpopulations of neurons, and this specificity was driven by the physical context of the task apparatus. The few neurons that were activated by both tasks did not have correlated choice preferences across tasks. Individual neurons in parietal cortex thus are not generalists but are rather specialists, active only in specific physical contexts.

RESULTS

We trained mice to perform two visual decision tasks while head fixed. In the first task ("T-maze task"), mice ran on an

This is an open access article under the CC BY license (http://creativecommons.org/licenses/by/4.0/).

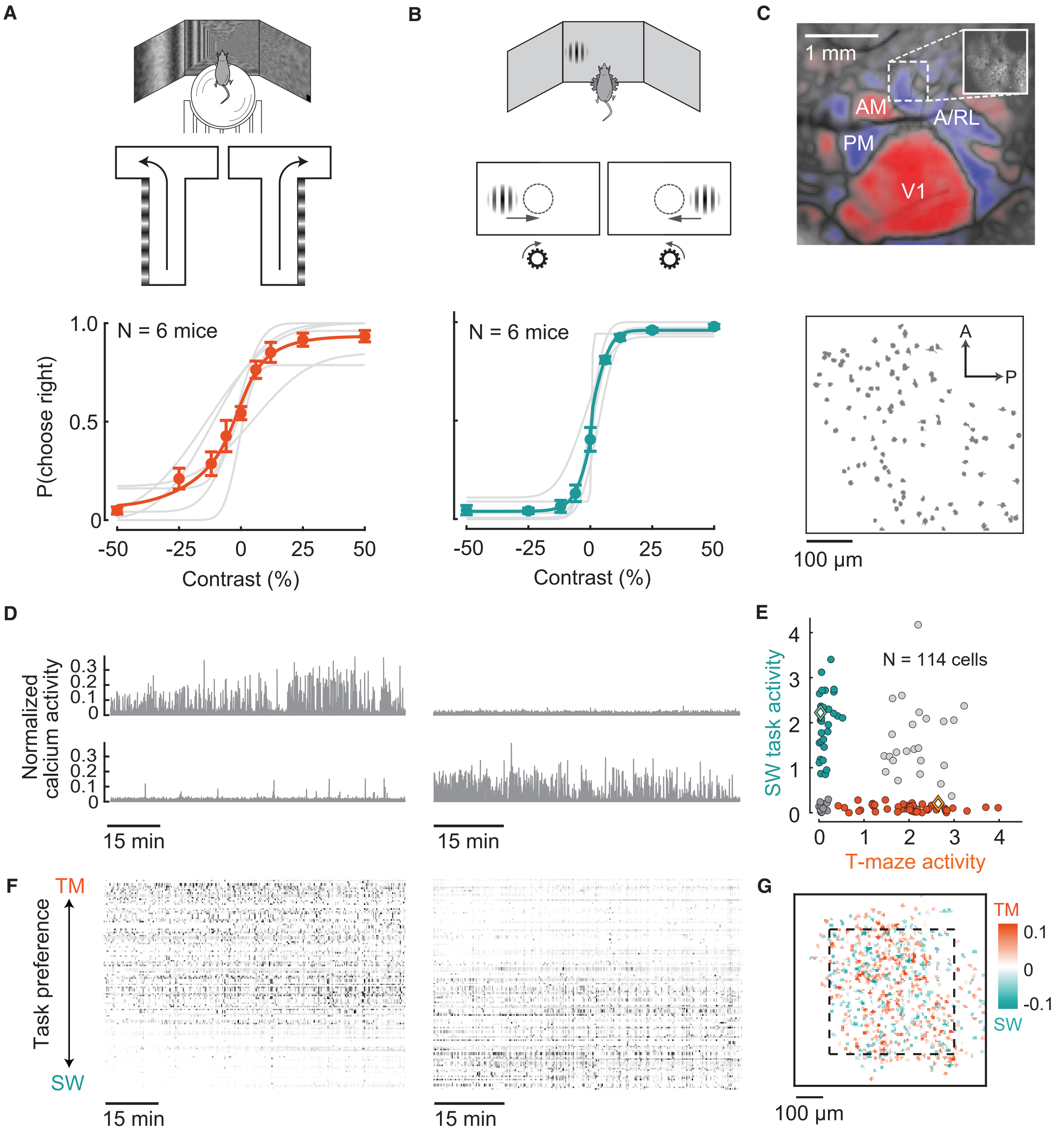

Figure 1. In mice performing two visual decision tasks, many parietal neurons are task specific

(A) Top: the T-maze task. Bottom: fraction of rightward choices versus contrast of stimuli on the left (negative) or on the right (positive). Dots and error bars show mean ± SD for n = 21 sessions in 6 mice. Curves show the fitted psychometric function for each mouse (gray) and averaged across mice (orange). Psychometric data for all sessions are in Figure S1

(B) Top: the steering-wheel task. Bottom: performance in the task of the same mice on the same days as (A).

(C) Top: map of visual cortical areas from wide-field imaging, showing the visual field sign of retinotopic areas (blue: negative; red: positive) and the field of view targeted for two-photon imaging (inset) from an example mouse. Bottom: outlines of the identified neurons in the field of view.

(D) Responses of two neurons from the example session, showing task-specific activity.

(E) Summary of activity (isolation distance) in the example session in the T-maze (TM) versus steering-wheel (SW) tasks, showing neurons that fired only in the T-maze task (orange), only in the steering-wheel task (blue), in both tasks (white), or in neither task (gray). Diamonds indicate the example neurons in (D).

(legend continued on next page)

CellPress
OPEN ACCESS

air-suspended styrofoam ball to navigate through a virtual T-maze and reported whether a grating was present on the left or right wall of the corridor by turning into the corresponding arm (Figure 1A, top) (Krumin et al., 2018). In the second task ("steering-wheel task"), mice sat on a platform, turned a steering wheel with their front paws, and reported whether a grating was on the left or right side by turning the wheel to bring the grating to the center (Figure 1B, top) (Burgess et al., 2017). To vary difficulty, the visual contrast on each trial was chosen from a range of values. We trained mice (n = 6) to perform both tasks consecutively on the same day. Mice typically performed hundreds of trials in each task. They performed well on both tasks, making more rightward choices with higher contrasts for stimuli on the right and more leftward choices with higher contrasts for stimuli on the left (Figures 1A and 1B, bottom).

We then used two-photon calcium imaging to record from the same population of parietal neurons in the two tasks. We targeted a parietal region anterior to the primary visual cortex and overlapping with visual areas A and RL (Gilissen et al., 2021; Hovde et al., 2019; Wang et al., 2020), identified by wide-field retinotopy (Figure 1C, top; Garrett et al., 2014; Sereno et al., 1994; Zhuang et al., 2017). We then imaged this region with a two-photon microscope (Figure 1C, inset) to record the activity of hundreds of parietal neurons simultaneously (Figure 1C, bottom). Mice were tested on both tasks in the same microscope.

Parietal neurons could participate in either task, but over half of them were task specific. Many neurons that were active during the T-maze task were inactive during the steering-wheel task, and vice versa (Figure 1D). To quantify active or inactive neurons, we summarized the activity of each neuron within each task using "isolation distance" (Stringer and Pachitariu, 2019), which characterizes a neuron's activity level relative to background neuropil fluorescence (STAR Methods). This measure captures intuitions of whether a neuron is active or inactive, and it is immune to minor differences in the magnitude of activity. Comparing this measure across tasks revealed that over half of the neurons with some activity were active only in the T-maze or only in the steering-wheel task, and only a minority were active in both tasks (Figure 1E). We obtained similar results with other measures of activity such as mean deconvolved firing rate (Figure S2). We used the difference in activity across tasks to sort neurons by their task preference (Figure 1F). Task-specific neurons seemed to intermix, with no obvious anatomical organization (Figure 1G).

If a neuron is active in one task, is it more or less likely to be active in the other task? Answering this question requires computing the correlation of the activities of neurons in the two tasks. For the cells in our sample, this correlation is negative (e.g., $r = -0.49$, $p < 1e{-6}$ in Figure 1E). However, the true correlation critically depends on the fraction of silent neurons, i.e., neurons that are inactive in both tasks (at the origin in Figure 1E). These silent neurons are largely missed by two-photon calcium imaging, which detects neurons based on their activity. Indeed, anatomical estimates of neural density (Keller et al., 2018) indicate that if all silent neurons were included, the correlation of activities would be highly positive ($r > 0.99$ in simulations), dominated by the high probability of a neuron being inactive in both tasks. Therefore, it would be hard to interpret correlations computed across tasks in this manner. However, our data allow an analysis that is stronger and independent of the number of silent neurons: comparing the same population across days.

Recording the same population across days revealed that task specificity was robust and repeatable. We imaged the same plane on a subsequent day and aligned cells recorded on both days using Suite2p (Pachitariu et al., 2017). We then compared each neuron's activity across days, within or across tasks (Figure 2A). Activity across days was highly correlated within tasks (Figure 2B) but negatively correlated or not significantly correlated across tasks (Figure 2C). Activity was significantly more similar within than across tasks (Figure 2D), whether considered for the T-maze ($p < 1e{-5}$) or steering-wheel task ($p < 0.001$, one-tailed t tests). Further, task specificity was independent of order: tasks were not always performed in the same order on different days (including sessions in Figures 2B–2D). In all, these results indicate that the task specificity shown by many parietal neurons is robust and stable across successive days (Figure 2E). Similar results were observed using mean firing rate as a measure of activity (Figure S2).

The task specificity of parietal neurons must then be attributable to repeatable factors that are inherent to each task. Some distinguishing factors might lie in the sensory context: although both tasks are based on vision, one involves visual scenes in virtual reality and the other involves a spatially isolated visual grating. Other distinguishing factors might lie in the physical context: the apparatus used to perform each task (an air-suspended ball versus a steering wheel), or the associated motor demands (running versus steering).

To investigate the role of physical context, we recorded the same neurons in each task apparatus while mice passively viewed a gray screen, and we found that neurons had similar specificity as in the task. Activity in each passive condition was similar to the activity in the respective task corresponding to the same apparatus and different from the activity in the other apparatus (Figure 3A). Activity was highly correlated within a physical context (Figure 3B), and it was uncorrelated or negatively correlated across contexts (Figure 3C). Correlations were significantly different across contexts but not within contexts (Figure 3D). This context specificity could not be accounted for by movement variables such as running (Figure S4), pupil diameter, or facial movements (Figure S5). For instance, when the mouse was in the T-maze task but did not run, activity still did not resemble the activity seen in the steering-wheel task (Figures S4E–S4G). In summary, parietal neurons showed context specificity for each task apparatus, and this specificity was not reducible to measured movements.

(F) Raster plots of neurons in an example session in the two tasks. Gray level denotes deconvolved calcium signal, *Z* scored. Neurons are sorted by relative task preference, i.e., the difference between the x and y values in (E).

(G) Anatomical distribution for the same example mouse, showing the overlay of ROIs over nine sessions, colored as in (E). Dashed square indicates a typical imaging field of view as in (C). Scale bar indicates extent of task selectivity (difference of task activity as in [E] normalized over the sum), averaged over sessions.

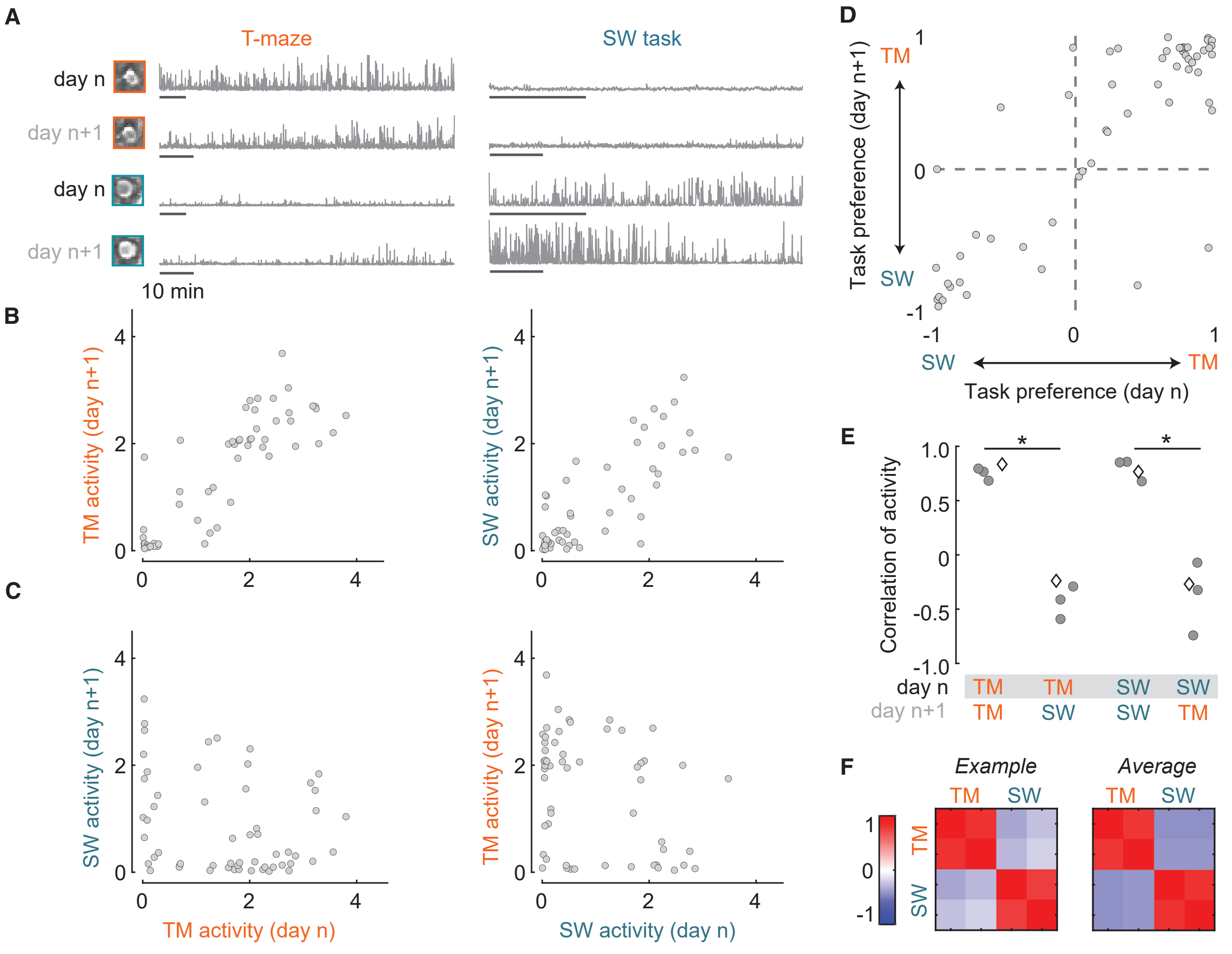

Figure 2. Task specificity is consistent across days

(A) Activity of two example neurons in the T-maze on consecutive days (left). Activity of the same neurons in the steering-wheel task across days (right). Insets show the mean images of each neuron in each session. Each bar represents 10 min.

(B) Comparison of activity within tasks across consecutive days, in the T-maze (left) or steering-wheel task (right). Correlations were positive in both cases ($r = 0.83$ and $r = 0.77$, $p \approx 0$, i.e., too small to measure).

(C) Same as in (B) but comparing activity across tasks. Correlations were negative (left: $r = -0.24$, $p = 0.08$) or not significant (right: $r = -0.27$, $p = 0.05$).

(D) Comparison of task preference (relative activity over tasks: positive for neurons preferring the T-maze task and negative for neurons preferring the steering-wheel task) for neurons imaged in two example consecutive days (N = 56 cells), showing significant correlation across days, $r = 0.84$, $p = 5e{-16}$. Correlations were also high in the other three pairs of days, with $r = 0.85$, 0.87, and 0.78.

(E) Summary from four pairs of days in three mice. Diamond illustrates the example pair of days from (B) and (C). Filled points indicate significant Spearman rank correlations at $p < 0.05$.

(F) Spearman rank correlation across all conditions, for the example pair of days in (B) and (C) (left) and the average over four pairs of days from (E) (right). Scale bar indicates Spearman rank correlation, rho (ρ).

To further confirm the role of physical context in determining task specificity, we trained two of our mice in a third, hybrid task, which combined the visual context of the T-maze (virtual corridor and rotational optic flow) with the physical context and motor demands of the steering wheel ("steering T-maze" task; Figure 4A). In some sessions, the mice were able to perform all three tasks consecutively, albeit with a smaller number of trials per task as expected due to satiation. In one such session, we could thus record the same neurons across all three tasks, and we found that activity was similar across tasks with the same physical context but dissimilar across tasks with different physical contexts (Figures 4B and 4C). In other sessions, where mice performed pairs of the three tasks, again we found that task participation of parietal neurons was correlated within but not across contexts (Figure 4D). Therefore, task specificity of parietal neurons is determined by physical context and not by visual context.

We then asked whether neurons that encode task variables of one task also encode variables of the other task. To evaluate each neuron's encoding of task-relevant events, we used an encoding model based on predictors such as stimulus onset,

CellPress
OPEN ACCESS

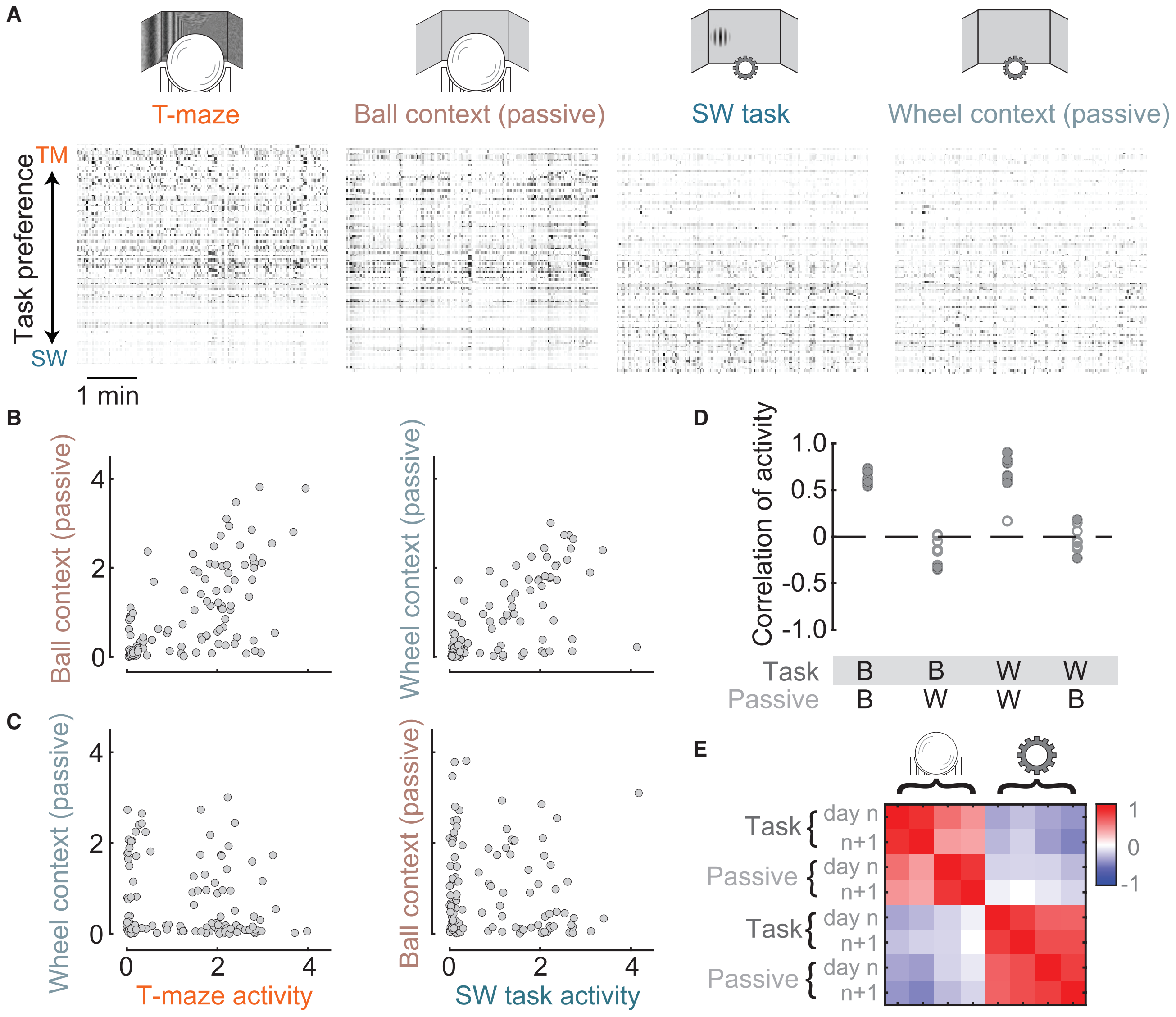

Figure 3. Task specificity is predictable by physical context in the absence of a task

(A) Raster plot of activity from neurons in an example session showing 5-min segments of activity in each task and in the corresponding passive condition. Gray level indicates normalized firing rate as in Figure 1. Left to right: T-maze, passive ball, steering-wheel task, and passive steering wheel.

(B) Comparison of activity for the same population of neurons across conditions with similar physical context, for the example session in Figures 2A–2C. Activity is highly correlated both within the ball context (left: $r = 0.63$, $p \approx 0$) and within the wheel context (right: $r = 0.65$, $p \approx 0$).

(C) Comparison of activity across different physical contexts for the same session. Activity is not significantly correlated (left: $r = -0.16$, $p = 0.09$; right: $r = -0.10$, $p = 0.28$).

(D) Summary of correlations of activity within and across physical contexts for 10 sessions where we recorded passive conditions. Filled circles indicate significant Spearman rank correlations. Correlations were different across but not within contexts, one-way ANOVA, $F(3,36) = 9.43$, $p = 1e-16$.

(E) Another pair of sessions where all four conditions were recorded on successive days. Color map and scale bar shows Spearman rank correlation, rho (ρ), of activity as before.

choice, and reward (Figure S6A; see STAR Methods). For each neuron, we applied this model separately to each task, obtaining good approximations of the neuron's activity (Figure S6B). To measure the degree to which a neuron encoded task-relevant variables in each task, we computed the cross-validated variance explained by the model based on those variables. These values were negatively correlated across tasks: neurons that were well predicted by task events in one task were poorly predicted by task events in the other (Figure S6C). In other words, the information encoded by parietal neurons is task specific: few neurons encoded task events in both physical contexts. This finding was typical across sessions (Figure S6D) and echoes what we found when comparing activity across tasks (Figure 1). Indeed, activity and encoding were closely related

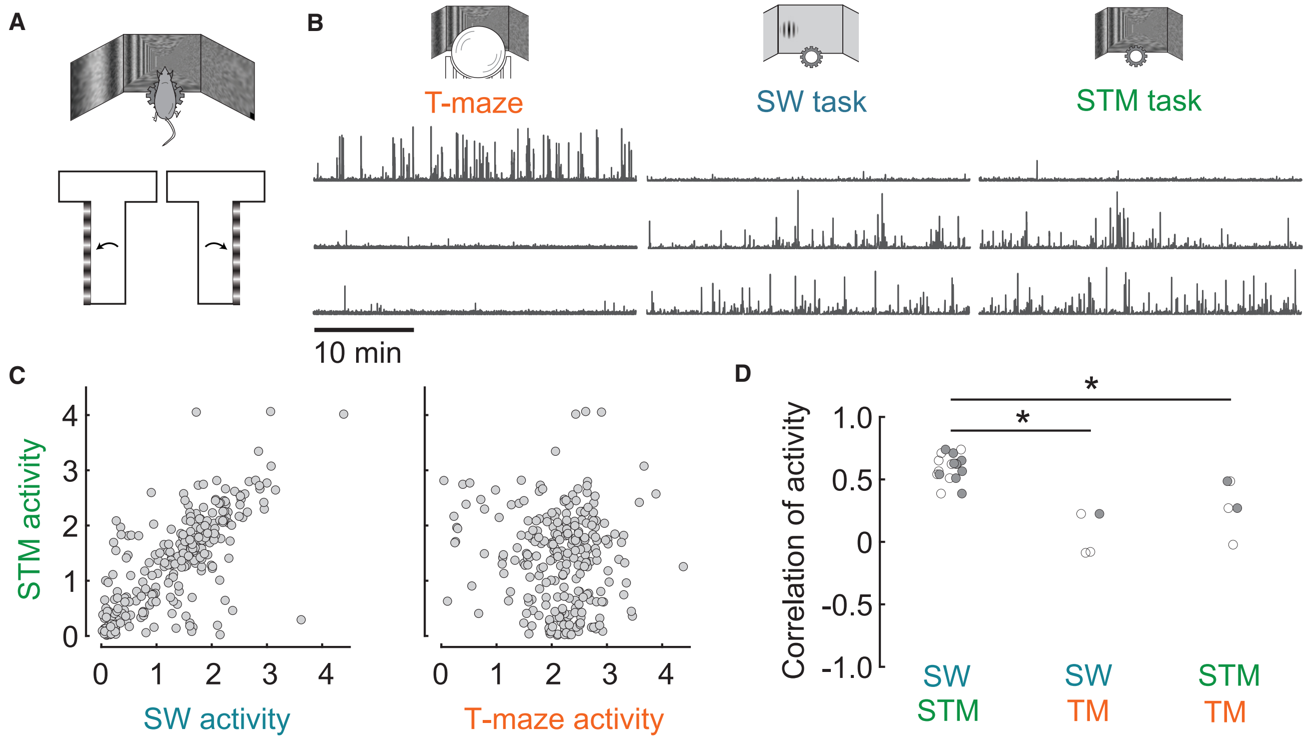

Figure 4. Activity in a hybrid task confirms the role of physical context

(A) The "steering T-maze" task (STM) combines the apparatus of the steering wheel with the visual scene of the T-maze in a fixed position along the corridor.

(B) Three example neurons from a session that included all three tasks.

(C) Activity of the same population of neurons across the steering-wheel and steering T-maze tasks (left: $r = 0.74$, $p \approx 0$) and across the T-maze and steering T-maze tasks (right: $r = -0.02$, $p = 0.72$) from the same session as (B).

(D) Summary of pairwise comparisons between the T-maze, steering-wheel, and hybrid tasks. SW versus hybrid: n = 9 sessions; SW versus TM: n = 3 sessions; TM versus hybrid: n = 3 sessions. Sessions were only included if mice performed all three tasks, and enough trials were acquired for each pair. A one-way ANOVA found significant group differences, $F(2,12) = 17.74$, $p = 0.0003$.

(Figure S6E). Neurons that were task specific in their activity were task specific in their encoding of task variables (Figure S6F). Neurons encoding task-relevant variables (>8% explained variance by each model) had strongly negative correlations in activity across tasks (median $r = -0.24$; Figure S6G). Once we excluded silent neurons, we confirmed that neurons inactive in one task tended to significantly encode signals related to the other task (Figure S6H).

Finally, we asked whether the few neurons active in both tasks had correlated choice preferences across tasks. The activity of these neurons was often well explained by both task-encoding models (explained variance was significantly correlated across tasks in 7/20 sessions, median $r = 0.08$ across sessions). We wondered whether this relationship occurred because they encoded the same abstract choice signals useful for both tasks. Separately, we could decode choice from neurons in each task (Figures S7A and S7B). As expected, significant choice selectivity was rare in the steering-wheel task: <10% of active neurons (Steinmetz et al., 2019; Zatka-Haas et al., 2021). Choice selectivity appeared to be more common in the T-maze, where it might reflect behavioral variables related to heading (Krumin et al., 2018). However, most importantly, choice preferences were not correlated across tasks (Figures S7C and S7D). Therefore, even in neurons engaged in both tasks, task-relevant signals such as choice preferences were specific to context.

DISCUSSION

By training the same mice in two visual decision tasks and recording from the same parietal neurons in both tasks, we discovered that most neurons are active during only one or the other task. This task specificity was reliable across successive days, indicating that it can be explained by factors inherent to each task. By recording in passive conditions and in a hybrid task, we established that such factors relate to each task's physical context. Task specificity was also evident when we characterized each neuron's activity by its encoding of task-relevant events. Lastly, context also influenced choice representations in the minority of parietal neurons that responded in both tasks. Therefore, physical context is a dominant factor that determines both a neuron's participation in a given task and the variables encoded in its activity.

Our findings are compatible with rodent studies that compared the activity of individual parietal neurons across sensory modalities. In our study, mice switched between two tasks that probed the same sensory modality but in different task

apparatus. In these conditions, many parietal neurons had different choice preferences across tasks. In other studies, rats switched between two tasks based on different sensory modalities but in the same task apparatus (Nikbakht et al., 2018; Raposo et al., 2014). In those conditions, parietal neurons had similar choice preferences across tasks. These findings are compatible with each other, and together they confirm that a parietal neuron's participation and encoding of choice variables depend not on sensory variables but rather on physical context.

The tasks we employed differ in multiple ways. For instance, in the T-maze task, mice sometimes make more motor errors. However, task difficulty is unlikely to explain our results because we find context specificity in the passive conditions, when there were no task demands. The tasks also differ in optic flow, which can modulate parietal activity (Diamanti et al., 2021; Minderer et al., 2019), but this is also unlikely to explain context specificity because in the passive conditions the screen was gray with no optic flow. Further, mice may hold different postures across tasks, and posture can modulate rodent parietal activity (Mimica et al., 2018). To further study the role of physical movements and body posture in task selectivity, future work should ideally carry out extensive video or EMG analysis.

Our hybrid task allowed us to test a key hypothesis about the role of physical context, but it is only one of many that could be devised. For instance, one could investigate whether it is the task apparatus or motor report that plays a special role, by varying the motor report within the same apparatus or using the same motor report in different physical contexts. Training mice in such variations of our tasks could be useful to reveal what aspects of physical context determine selectivity. Indeed, in our study, "physical context" is used generally to indicate any conjunction of motor, sensory, or cognitive features unique to task apparatus.

These findings emphasize the value of sampling multiple behaviors in the same neuronal population. During any one experiment, most neurons are silent, as suggested by anatomy (Keller et al., 2018) and physiology (Thompson and Best, 1989). Our results show that apparently silent neurons in one physical context become active when mice are in a different context. Presumably yet another population would have been active in a third context. This stark dependence of activity on physical context might apply brainwide, beyond parietal cortex. Notably, different hippocampal neurons are active in different spatial contexts (Guzowski et al., 1999; Kubie and Ranck, 1983; Leutgeb et al., 2005), and the amygdala also may have spatial context specificity (Gründemann et al., 2019).

Context-specific populations could be a valuable signal for downstream regions to perform efficient, context-specific computations. Neurons in the same parietal population project to different targets and convey highly specific sensorimotor signals (Hwang et al., 2019; Itokazu et al., 2018). Perhaps context-specific neurons project to different areas for different purposes. Altogether, engaging distinct subpopulations in different contexts might help animals make the right choices in the right contexts.

STAR★METHODS

Detailed methods are provided in the online version of this paper and include the following:

- KEY RESOURCES TABLE
- RESOURCE AVAILABILITY
  - Lead contact
  - Materials availability
  - Data and code availability
- EXPERIMENTAL MODEL AND SUBJECT DETAILS
- METHOD DETAILS
  - Surgery
  - Habituation
  - Behavioral training and testing
  - Neural recordings
  - Data analysis

SUPPLEMENTAL INFORMATION

Supplemental information can be found online at https://doi.org/10.1016/j.neuron.2022.07.017.

ACKNOWLEDGMENTS

We thank Laura Funnell, Miles Wells, and Hamish Forrest for assistance with mouse training; Charu Reddy for laboratory support; and Samuel Picard for suggestions on the manuscript. This work was supported by the Wellcome Trust through a PhD studentship to J.J.L. (grant 109004) and joint Investigator Award to K.D.H. and M.C. (grants 205093 and 223144). M.C. holds the GlaxoSmithKline/Fight for Sight Chair in Visual Neuroscience.

AUTHOR CONTRIBUTIONS

Conceptualization, J.J.L., M.K., M.C., and K.D.H.; methodology, J.J.L. and M.K.; software, J.J.L. and M.K.; formal analysis, J.J.L. and M.K.; investigation, J.J.L.; data curation, J.J.L.; writing – original draft, J.J.L. and M.C.; writing – review & editing, J.J.L., M.K., M.C., and K.D.H.; supervision, M.C. and K.D.H.; funding acquisition, J.J.L., K.D.H., and M.C.

DECLARATION OF INTERESTS

M.C. is a member of *Neuron*'s advisory board.

Received: December 21, 2020
Revised: January 16, 2022
Accepted: July 15, 2022
Published: August 12, 2022

REFERENCES

Bhagat, J., Wells, M.J., Harris, K.D., Carandini, M., and Burgess, C.P. (2020). Rigbox: an open-source toolbox for probing neurons and behavior. eNeuro *7*. ENEURO.0406-19.2020. https://doi.org/10.1523/ENEURO.0406-19.2020.

Britten, K.H., Newsome, W.T., Shadlen, M.N., Celebrini, S., and Movshon, J.A. (1996). A relationship between behavioral choice and the visual responses of neurons in macaque MT. Vis. Neurosci. *13*, 87–100. https://doi.org/10.1017/s095252380000715x.

Burgess, C.P., Lak, A., Steinmetz, N.A., Zatka-Haas, P., Bai Reddy, C., Jacobs, E.A.K., Linden, J.F., Paton, J.J., Ranson, A., Schröder, S., et al. (2017). High-yield methods for accurate two-alternative visual psychophysics in head-fixed mice. Cell Rep. *20*, 2513–2524. https://doi.org/10.1016/j.celrep.2017.08.047.

Busse, L., Ayaz, A., Dhruv, N.T., Katzner, S., Saleem, A.B., Schölvinck, M.L., Zaharia, A.D., and Carandini, M. (2011). The detection of visual contrast in the behaving mouse. J. Neurosci. *31*, 11351–11361. https://doi.org/10.1523/JNEUROSCI.6689-10.2011.

Chen, J.L., Carta, S., Soldado-Magraner, J., Schneider, B.L., and Helmchen, F. (2013a). Behaviour-dependent recruitment of long-range projection

neurons in somatosensory cortex. Nature *499*, 336–340. https://doi.org/10.1038/nature12236.

Chen, J.L., Margolis, D.J., Stankov, A., Sumanovski, L.T., Schneider, B.L., and Helmchen, F. (2015). Pathway-specific reorganization of projection neurons in somatosensory cortex during learning. Nat. Neurosci. *18*, 1101–1108. https://doi.org/10.1038/nn.4046.

Chen, L.L., Lin, L.H., Green, E.J., Barnes, C.A., and McNaughton, B.L. (1994). Head-direction cells in the rat posterior cortex. I. Anatomical distribution and behavioral modulation. Exp. Brain Res. *101*, 8–23. https://doi.org/10.1007/bf00243212.

Chen, T.W., Wardill, T.J., Sun, Y., Pulver, S.R., Renninger, S.L., Baohan, A., Schreiter, E.R., Kerr, R.A., Orger, M.B., Jayaraman, V., et al. (2013b). Ultrasensitive fluorescent proteins for imaging neuronal activity. Nature *499*, 295–300. https://doi.org/10.1038/nature12354.

Diamanti, E.M., Reddy, C.B., Schröder, S., Muzzu, T., Harris, K.D., Saleem, A.B., and Carandini, M. (2021). Spatial modulation of visual responses arises in cortex with active navigation. eLife *10*, e63705. https://doi.org/10.7554/eLife.63705.

Dipoppa, M., Ranson, A., Krumin, M., Pachitariu, M., Carandini, M., and Harris, K.D. (2018). Vision and locomotion shape the interactions between neuron types in mouse visual cortex. Neuron *98*. 602.e8–615.e8. https://doi.org/10.1016/j.neuron.2018.03.037.

Friedrich, J., Zhou, P., and Paninski, L. (2017). Fast online deconvolution of calcium imaging data. PLoS Comput. Biol. *13*, e1005423. https://doi.org/10.1371/journal.pcbi.1005423.

Garrett, M.E., Nauhaus, I., Marshel, J.H., and Callaway, E.M. (2014). Topography and areal organization of mouse visual cortex. J. Neurosci. *34*, 12587–12600. https://doi.org/10.1523/JNEUROSCI.1124-14.2014.

Gilissen, S.R.J., Farrow, K., Bonin, V., and Arckens, L. (2021). Reconsidering the border between the visual and posterior parietal cortex of mice. Cereb. Cortex *31*, 1675–1692. https://doi.org/10.1093/cercor/bhaa318.

Gnadt, J.W., and Andersen, R.A. (1988). Memory related motor planning activity in posterior parietal cortex of macaque. Exp. Brain Res. *70*, 216–220. https://doi.org/10.1007/bf00271862.

Gründemann, J., Bitterman, Y., Lu, T., Krabbe, S., Grewe, B.F., Schnitzer, M.J., and Lüthi, A. (2019). Amygdala ensembles encode behavioral states. Science *364*, eaav8736. https://doi.org/10.1126/science.aav8736.

Guzowski, J.F., McNaughton, B.L., Barnes, C.A., and Worley, P.F. (1999). Environment-specific expression of the immediate-early gene Arc in hippocampal neuronal ensembles. Nat. Neurosci. *2*, 1120–1124. https://doi.org/10.1038/16046.

Hanks, T.D., Kopec, C.D., Brunton, B.W., Duan, C.A., Erlich, J.C., and Brody, C.D. (2015). Distinct relationships of parietal and prefrontal cortices to evidence accumulation. Nature *520*, 220–223. https://doi.org/10.1038/nature14066.

Harvey, C.D., Coen, P., and Tank, D.W. (2012). Choice-specific sequences in parietal cortex during a virtual-navigation decision task. Nature *484*, 62–68. https://doi.org/10.1038/nature10918.

Hovde, K., Gianatti, M., Witter, M.P., and Whitlock, J.R. (2019). Architecture and organization of mouse posterior parietal cortex relative to extrastriate areas. Eur. J. Neurosci. *49*, 1313–1329. https://doi.org/10.1111/ejn.14280.

Huang, L., Ledochowitsch, P., Knoblich, U., Lecoq, J., Murphy, G.J., Reid, R.C., de Vries, S.E., Koch, C., Zeng, H., Buice, M.A., et al. (2021). Relationship between simultaneously recorded spiking activity and fluorescence signal in GCaMP6 transgenic mice. eLife *10*, e51675. https://doi.org/10.7554/eLife.51675.

Hwang, E.J., Link, T.D., Hu, Y.Y., Lu, S., Wang, E.H., Lilascharoen, V., Aronson, S., O'Neil, K., Lim, B.K., and Komiyama, T. (2019). Corticostriatal flow of action selection bias. Neuron *104*. 1126.e6–1140.e6. https://doi.org/10.1016/j.neuron.2019.09.028.

International Brain Laboratory, Aguillon-Rodriguez, V., Angelaki, D., Bayer, H., Bonacchi, N., Carandini, M., Cazettes, F., Chapuis, G., Churchland, A.K., Dan, Y., et al. (2021). Standardized and reproducible measurement of decision-making in mice. eLife *10*, e63711. https://doi.org/10.7554/eLife.63711.

Itokazu, T., Hasegawa, M., Kimura, R., Osaki, H., Albrecht, U.R., Sohya, K., Chakrabarti, S., Itoh, H., Ito, T., Sato, T.K., and Sato, T.R. (2018). Streamlined sensory motor communication through cortical reciprocal connectivity in a visually guided eye movement task. Nat. Commun. *9*, 338. https://doi.org/10.1038/s41467-017-02501-4.

Keller, D., Erö, C., and Markram, H. (2018). Cell densities in the mouse brain: a systematic review. Front. Neuroanat. *12*, 83. https://doi.org/10.3389/fnana.2018.00083.

Kleiner, M., Brainard, D., and Pelli, D. (2007). What's new in psychtoolbox-3. Perception (suppl) *36*, 1–16.

Krumin, M., Lee, J.J., Harris, K.D., and Carandini, M. (2018). Decision and navigation in mouse parietal cortex. eLife *7*, e42583. https://doi.org/10.7554/eLife.42583.

Kubie, J., and Ranck, J.B. (1983). Sensory-behavioral correlates in individual hippocampus neurons in three situations: space and context. In Neurobiology of the Hippocampus, W. Seifert, ed. (Academic Press).

Leutgeb, J.K., Leutgeb, S., Treves, A., Meyer, R., Barnes, C.A., McNaughton, B.L., Moser, M.B., and Moser, E.I. (2005). Progressive transformation of hippocampal neuronal representations in "morphed" environments. Neuron *48*, 345–358. https://doi.org/10.1016/j.neuron.2005.09.007.

Meister, M.L., Hennig, J.A., and Huk, A.C. (2013). Signal multiplexing and single-neuron computations in lateral intraparietal area during decision-making. J. Neurosci. *33*, 2254–2267. https://doi.org/10.1523/JNEUROSCI.2984-12.2013.

Mimica, B., Dunn, B.A., Tombaz, T., Bojja, V.P.T., and Whitlock, J.R. (2018). Efficient cortical coding of 3D posture in freely behaving rats. Science *362*, 584–589. https://doi.org/10.1126/science.aau2013.

Minderer, M., Brown, K.D., and Harvey, C.D. (2019). The spatial structure of neural encoding in mouse posterior cortex during navigation. Neuron *102*. 232.e11–248.e11. https://doi.org/10.1016/j.neuron.2019.01.029.

Mohan, K., Zhu, O., and Freedman, D.J. (2021). Interaction between neuronal encoding and population dynamics during categorization task switching in parietal cortex. Neuron *109*. 700.e4–712.e4. https://doi.org/10.1016/j.neuron.2020.11.022.

Nikbakht, N., Tafreshiha, A., Zoccolan, D., and Diamond, M.E. (2018). Supralinear and supramodal integration of visual and tactile signals in rats: psychophysics and neuronal mechanisms. Neuron *97*. 626.e8–639.e8. https://doi.org/10.1016/j.neuron.2018.01.003.

Nitz, D.A. (2006). Tracking route progression in the posterior parietal cortex. Neuron *49*, 747–756. https://doi.org/10.1016/j.neuron.2006.01.037.

Nitz, D.A. (2012). Spaces within spaces: rat parietal cortex neurons register position across three reference frames. Nat. Neurosci. *15*, 1365–1367. https://doi.org/10.1038/nn.3213.

Olsen, G.M., and Witter, M.P. (2016). Posterior parietal cortex of the rat: architectural delineation and thalamic differentiation. J. Comp. Neurol. *524*, 3774–3809. https://doi.org/10.1002/cne.24032.

Pachitariu, M., Stringer, C., Schröder, S., Rossi, L.F., Dipoppa, M., Carandini, M., and Harris, K.D. (2017). Suite2p: beyond 10,000 neurons with standard two-photon microscopy. Preprint at bioRxiv. https://doi.org/10.1101/061507.

Park, I.M., Meister, M.L., Huk, A.C., and Pillow, J.W. (2014). Encoding and decoding in parietal cortex during sensorimotor decision-making. Nat. Neurosci. *17*, 1395–1403. https://doi.org/10.1038/nn.3800.

Pinto, L., Rajan, K., DePasquale, B., Thiberge, S.Y., Tank, D.W., and Brody, C.D. (2019). Task-dependent changes in the large-scale dynamics and necessity of cortical regions. Neuron *104*. 810.e9–824.e9. https://doi.org/10.1016/j.neuron.2019.08.025.

Pologruto, T.A., Sabatini, B.L., and Svoboda, K. (2003). ScanImage: flexible software for operating laser scanning microscopes. Biomed. Eng. OnLine *2*, 13. https://doi.org/10.1186/1475-925X-2-13.

Ramesh, R.N., Burgess, C.R., Sugden, A.U., Gyetvan, M., and Andermann, M.L. (2018). Intermingled ensembles in visual association cortex encode

CellPress
OPEN ACCESS

stimulus identity or predicted outcome. Neuron *100*. 900.e9–915.e9. https://doi.org/10.1016/j.neuron.2018.09.024.

Raposo, D., Kaufman, M.T., and Churchland, A.K. (2014). A category-free neural population supports evolving demands during decision-making. Nat. Neurosci. *17*, 1784–1792. https://doi.org/10.1038/nn.3865.

Sereno, M.I., McDonald, C.T., and Allman, J.M. (1994). Analysis of retinotopic maps in extrastriate cortex. Cereb. Cortex *4*, 601–620. https://doi.org/10.1093/cercor/4.6.601.

Shadlen, M.N., and Newsome, W.T. (2001). Neural basis of a perceptual decision in the parietal cortex (area LIP) of the rhesus monkey. J. Neurophysiol. *86*, 1916–1936. https://doi.org/10.1152/jn.2001.86.4.1916.

Snyder, L.H., Batista, A.P., and Andersen, R.A. (1997). Coding of intention in the posterior parietal cortex. Nature *386*, 167–170. https://doi.org/10.1038/386167a0.

Steinmetz, N.A., Buetfering, C., Lecoq, J., Lee, C.R., Peters, A.J., Jacobs, E.A.K., Coen, P., Ollerenshaw, D.R., Valley, M.T., de Vries, S.E.J., et al. (2017). Aberrant cortical activity in multiple GCaMP6-expressing transgenic mouse lines. eNeuro *4*. ENEURO.0207-17.2017. https://doi.org/10.1523/ENEURO.0207-17.2017.

Steinmetz, N.A., Zatka-Haas, P., Carandini, M., and Harris, K.D. (2019). Distributed coding of choice, action and engagement across the mouse brain. Nature *576*, 266–273. https://doi.org/10.1038/s41586-019-1787-x.

Stringer, C. (2018). Discovering Structure in Multi-Neuron Recordings Through Network Modelling (University College London).

Stringer, C., and Pachitariu, M. (2019). Computational processing of neural recordings from calcium imaging data. Curr. Opin. Neurobiol. *55*, 22–31. https://doi.org/10.1016/j.conb.2018.11.005.

Stringer, C., Pachitariu, M., Steinmetz, N., Reddy, C.B., Carandini, M., and Harris, K.D. (2019). Spontaneous behaviors drive multidimensional, brainwide activity. Science *364*, 255. https://doi.org/10.1126/science.aav7893.

Thompson, L.T., and Best, P.J. (1989). Place cells and silent cells in the hippocampus of freely-behaving rats. J. Neurosci. *9*, 2382–2390. https://doi.org/10.1523/jneurosci.09-07-02382.1989.

Wang, Q., Ding, S.L., Li, Y., Royall, J., Feng, D., Lesnar, P., Graddis, N., Naeemi, M., Facer, B., Ho, A., et al. (2020). The Allen mouse brain common coordinate framework: a 3D reference atlas. Cell *181*. 936.e20–953.e20. https://doi.org/10.1016/j.cell.2020.04.007.

Wekselblatt, J.B., Flister, E.D., Piscopo, D.M., and Niell, C.M. (2016). Large-scale imaging of cortical dynamics during sensory perception and behavior. J. Neurophysiol. *115*, 2852–2866. https://doi.org/10.1152/jn.01056.2015.

Whitlock, J.R. (2017). Posterior parietal cortex. Curr. Biol. *27*, R691–R695. https://doi.org/10.1016/j.cub.2017.06.007.

Whitlock, J.R., Pfuhl, G., Dagslott, N., Moser, M.B., and Moser, E.I. (2012). Functional split between parietal and entorhinal cortices in the rat. Neuron *73*, 789–802. https://doi.org/10.1016/j.neuron.2011.12.028.

Wilber, A.A., Clark, B.J., Forster, T.C., Tatsuno, M., and McNaughton, B.L. (2014). Interaction of egocentric and world-centered reference frames in the rat posterior parietal cortex. J. Neurosci. *34*, 5431–5446. https://doi.org/10.1523/JNEUROSCI.0511-14.2014.

Zatka-Haas, P., Steinmetz, N.A., Carandini, M., and Harris, K.D. (2021). Sensory coding and the causal impact of mouse cortex in a visual decision. eLife *10*, e63163. https://doi.org/10.7554/eLife.63163.

Zhang, C.Y., Aflalo, T., Revechkis, B., Rosario, E.R., Ouellette, D., Pouratian, N., and Andersen, R.A. (2017). Partially mixed selectivity in human posterior parietal association cortex. Neuron *95*. 697.e4–708.e4. https://doi.org/10.1016/j.neuron.2017.06.040.

Zhuang, J., Ng, L., Williams, D., Valley, M., Li, Y., Garrett, M., and Waters, J. (2017). An extended retinotopic map of mouse cortex. eLife *6*, e18372. https://doi.org/10.7554/eLife.18372.

STAR★METHODS

KEY RESOURCES TABLE

REAGENT or RESOURCE	SOURCE	IDENTIFIER
Deposited data		
Processed two-photon imaging data and behavioral data	This paper	https://osf.io/vjpw4/
Experimental models: Organisms/strains		
Mouse: tetO-6GCaMP6s	https://www.jax.org/strain/024742	RRID:IMSR_JAX:024742
Mouse: CaMKII-tTA	https://www.jax.org/strain/007004	RRID:IMSR_JAX:007004
Mouse: Ai95(RCL-GCaMP6f)-D	https://www.jax.org/strain/028865	RRID:IMSR_JAX:028865
Mouse: Vglut1-IRES2-Cre-D	https://www.jax.org/strain/023527	RRID:IMSR_JAX:023527
Software and algorithms		
MATLAB	MathWorks	https://www.mathworks.com
ScanImage	Pologruto et al. (2003)	https://vidriotechnologies.com/
Psychophysics Toolbox	Kleiner et al. (2007)	http://psychtoolbox.org/
Rigbox	Bhagat et al. (2020)	https://github.com/cortex-lab/Rigbox
Suite2p (MATLAB implementation)	Pachitariu et al. (2017)	https://github.com/cortex-lab/Suite2P/
OASIS spike deconvolution (MATLAB implementation)	Friedrich et al. (2017)	https://github.com/zhoupc/OASIS_matlab
EyeTracking	This paper	https://github.com/mkrumin/EyeTracking
FaceMap (MATLAB implementation)	Stringer et al. (2019)	https://github.com/MouseLand/facemap
Steering wheel movement detection	Steinmetz et al. (2019)	https://github.com/cortex-lab/wheelAnalysis/tree/master/+wheel
Isolation distance estimation	Stringer and Pachitariu (2019)	N/A
Combined conditions choice probability	Steinmetz et al. (2019)	N/A
Matlab scripts to analyze our data and reconstruct the figures	This paper	https://osf.io/vjpw4/
Other		
Thorlabs B-Scope	Thorlabs	N/A
PCO Edge 5.5 sCMOS camera	PCO	N/A

RESOURCE AVAILABILITY

Lead contact

Requests for further information should be directed to the lead contact, Julie J. Lee, New York University, juliejlee@nyu.edu.

Materials availability

The materials used in this study are available commercially.

Data and code availability

Processed data from this study are available in a public repository as indicated in the key resources table. Likewise, analysis code for the main results is publicly available as indicated in the key resources table. Raw data is available from the lead contact upon request, subject to file size for data transfer constraints. Any additional data or code may be obtained from the lead contact upon request.

EXPERIMENTAL MODEL AND SUBJECT DETAILS

Mice were bred from transgenic lines that expressed the genetically encoded calcium indicator GCaMP in excitatory neurons. One mouse (female) expressed GCaMP6f in glutamatergic neurons (double transgenic Ai95(RCL-GCaMP6f)-D x Slc17a7-IRES2-Cre-D). Five mice (2 female, 3 male) expressed GCaMP6s in Camk2a-positive (excitatory) neurons (double transgenic tetO-6GCaMP6s x

Camk2a-tTA; Wekselblatt et al., 2016) with GCaMP. Neither line was found to display aberrant activity in the form of interictal spikes (Steinmetz et al., 2017). Mice were 7-18 weeks old (median 11.5 weeks) at the time of surgery and experiments were carried out until they were 20-38 weeks of age (median 32 weeks).

METHOD DETAILS

All experiments were conducted in accordance with the UK Animals Scientific Procedures Act (1986) following Home Office Guidelines.

Surgery

Surgical procedures were performed under aseptic conditions and under general anesthesia. Mice were anesthetized with isoflurane (Merial) at 3–5% for induction, and 0.75–1.5% subsequently. Body temperature was maintained at 37 °C using a heating pad. Carprofen (5 mg/kg, Rimadyl, Pfizer) was administered subcutaneously for systemic analgesia, and dexamethasone (0.5 mg/kg, Colvasone, Norbrook) was administered to prevent brain swelling. The scalp was shaved and disinfected, and a local analgesic was applied prior to the incision (Lidocaine, 5% ointment, TEVA UK; or intradermal injection, 6 mg/kg, Hameln Pharmaceuticals Ltd). The eyes were covered with eye-protective gel (Viscotears, Alcon; or Chloramphenicol, Martindale Ltd). The mouse was positioned in a stereotaxic frame (Lidocaine ointment was applied to the ear bars), the skin covering and surrounding the area of interest was removed, and the skull was cleaned of connective tissue. A custom headplate was positioned above the area of interest and attached to the bone with Superbond C and B (Sun Medical). Then, a round craniotomy (3–4 mm diameter) was made over the right posterior cortex with a fine-tipped diamond drill and/or a biopsy punch (Kai Medical). The craniotomy was centered at stereotaxic coordinates -2 mm Posterior to Bregma and 2 mm Lateral. The craniotomy was covered with glass (a 5-mm diameter outer coverslip glued to a 4-mm inner coverslip). A circular metal headplate of 7 mm radius was attached with dental cement. Following surgery, mice were placed in a heated container until they were ambulatory. Mice were then given Carprieve in water as an analgesic and were given at least three days to recover.

Habituation

Following recovery, mice were habituated gradually to the apparatus. They were first handled in their home cage, then gradually introduced to longer periods of head fixation. Once they were comfortable on the rig, two-photon imaging and wide-field retinotopy was acquired to ensure adequate imaging quality. If these criteria were passed, mice were water restricted so that water could be used as a reward. Body weight was monitored to ensure mice maintained at least 80% of their initial body weight. A minimum water allowance of 40 mL/kg per day was provided. If a mouse did not receive this daily allowance when performing the tasks, the rest of the fluids were delivered afterwards in the form of water or hydrogel. After at least two days of water restriction to ensure a stable weight and no adverse effects, mice were slowly introduced to elements of the task.

Behavioral training and testing

Apparatus

The mouse was head-fixed and surrounded by three computer screens (Iiyama ProLite E1980SD) at right angles, with the central screen ~20 cm away. The screens spanned ~270 deg horizontally and 70-75 deg vertically and refreshed at 60 Hz. Fresnel lenses were mounted in front of the screens to correct for aberrations in luminance and contrast at steeper viewing angles, covered with a scattering window film to prevent specular reflections (Burgess et al., 2017). A nearby speaker played auditory stimuli associated with task events, i.e., onset tones, reward tones, and incorrect noise bursts. A water spout was positioned near the mouth. Water delivery was controlled by a valve muffled in a block of foam, which retained an audible click on reward delivery.

Training protocol

Head-fixed mice were trained on two visual detection tasks for water reward, under the same imaging rig. One involved virtual navigation by running on a Styrofoam ball, and the other involved turning a steering wheel to move a visual grating. Mice were usually trained to asymptotic performance on one task before they were introduced to the other. Some mice started with the T-maze and others started with the steering-wheel task. Both tasks involved a vertical grating on either the left (overlapping with -30 deg azimuth) or right (overlapping with +30 deg azimuth) side of the visual field, at central elevation. In both tasks, mice had to orient in the same direction to bring the stimulus to the center of their visual field to make a correct response. Orienting in the opposite direction, pushing the stimulus away from the center, was an incorrect response. On each trial, a grating was uniformly randomly chosen among 0%, 6%, 12%, 25% and 50% contrasts. Mice received a reward (2 μl of water) for correct choices and a short auditory noise burst for incorrect choices. Both tasks shared task cues such as the onset tone, reward tone, and an initial open-loop interval of at least 200 ms when movements of the apparatus did not move the stimulus. A gray screen was presented during the inter-trial interval. To help with shaping, in early training, contrasts were initially restricted to including only high contrast subsets and 100% contrast, and mice received larger rewards (3-4 μl). Some mice received sucrose water in training to make the reward more appealing. A shorter inter-trial interval was also employed to prevent disengagement by waiting too long in between trials.

Steering-wheel task

The steering-wheel task is described in Burgess et al. (2017). In the task, mice sit on a raised platform within a "half-pipe" well. Their forepaws rest on a Lego steering wheel which they can rotate in a clockwise or counterclockwise direction. Stimulus presentation was delivered using the ''Rigbox'' package (Bhagat et al., 2020). At the beginning of a trial, a visual grating in a Gaussian window (a Gabor stimulus) appears on the left or right side of the screen at ± 30 deg azimuth. The mouse can move the wheel to move the grating along the horizontal direction, either an additional 30 deg to the periphery (± 60 deg azimuth) or 30 deg to the center (0 deg azimuth). The wheel was allowed to move immediately, but for the first 200 or 500 ms the stimulus was immobile regardless of wheel movements (open loop). Stimulus size was 9 deg (σ of the Gaussian envelope) in initial experiments and 20 deg in later experiments. The inter-trial interval was typically 0.5-3 s, and trials were terminated only if the mouse did not respond within 60 s. Wheel movements were detected using the ''findWheelMoves3'' MATLAB script (Steinmetz et al., 2019).

T-maze task

The T-maze task is described in Krumin et al. (2018). In the task, mice run on a Styrofoam ball (20 cm diameter) that is lightly suspended by pressurized air. Movements of the ball were measured using two optical computer mice to control a virtual reality scene. Mice control the ball by running on it. The rotation around the horizontal left-right axis (pitch) was responsible for forward movement in virtual reality, and the rotation of the ball around the vertical axis (yaw) was responsible for turning in virtual reality. The lateral displacement of the ball (rotation around the horizontal front-back axis, roll) was ignored. At the start of each trial, mice are shown a virtual reality T-maze with a long corridor, and two directions to turn at the end perpendicular to the initial corridor. The visual stimulus (grating) was displayed on the entire left or right wall of the initial corridor. The open-loop interval lasted 200 ms. To make their choice, mice needed to run down the initial corridor and turn left or right down the arms of the T, receiving a reward for a correct choice or auditory white noise burst for an incorrect choice. Trials were separated by at least 1.5-3 s. The virtual scene was controlled using a custom virtual reality engine implemented in MATLAB accessing OpenGL through the Psychophysics Toolbox (Kleiner et al., 2007). The initial corridor was 110 cm long including the juncture of the T, and 20 cm wide, with the two arms spanning 60 cm in width, i.e., an additional 20 cm to the left and right. Noise textures were displayed at 20% contrast on the walls and at 40% on the floor. The grating was superimposed additively on the noise texture.

Testing protocol

Mice were tested serially in two blocks, with full performance of one task before performance on the other. This was necessary to switch the apparatus across tasks. The gap between tasks was usually no more than a few minutes. To obtain similar numbers of trials in each task, mice were switched to the other task when they reached approximately half of their daily water allowance, typically 100-300 trials depending on performance. The second task was stopped when mice reached their minimum daily water allowance, or stopped performing trials or made many consecutive errors, whichever came first. Mice typically performed 100-300 trials/task/session with a duration of 20-60 min per task. Mice were often tested on the T-maze first, but sometimes performed the steering-wheel task first. All mice included in the dataset had fully learned and reached asymptotic performance in both tasks. To plot behavioral performance, psychometric curves were fitted using maximum likelihood estimation (Busse et al., 2011).

Passive conditions

In the same imaging session as the tasks, we occasionally imaged the same neurons in a passive condition on the same apparatus as either task (i.e., the spherical treadmill or steering wheel apparatus) but with no task, and a gray screen. These passive conditions usually followed each task, for 5-60 min, but occasionally were included before each task instead.

Hybrid task

In the hybrid task, the mouse uses the apparatus of the steering-wheel task but views the virtual scene of the T-maze. The mouse was placed at a fixed location 50 cm into the virtual corridor and started the trial looking straight ahead (θ = 0 deg). Turning the steering wheel rotated the view angle (θ). The gain of the steering wheel was matched to the original steering-wheel task, such that a choice required the same amount of turning in both tasks.

Neural recordings

Widefield imaging

To identify parietal cortex, we mapped known retinotopic areas by presenting sparse visual noise under widefield imaging. The protocol for widefield imaging followed standard procedure from the literature (e.g., Garrett et al., 2014; Zhuang et al., 2017). The entire 4 mm window was imaged under a wide field macroscope with dual illumination using a sCMOS camera (PCO Edge 5.5). Illumination was generated using an LED (Cairn OptoLED) alternating frames of violet (405 nm, excitation filter ET405/20x) and blue (470nm, excitation filter ET470/40x) light (at 35 Hz each) to capture calcium-dependent fluorescence and calcium-independent hemodynamic activity respectively. The visual stimulus consisted of black and white squares appearing asynchronously on a gray background. Widefield imaging movies were processed to filter out potential hemodynamic artefacts at the heartbeat frequency 7-13 Hz. Then a visual field sign map (Garrett et al., 2014; Sereno et al., 1994) was generated by taking the difference (sine of the angle) between the gradients of the azimuth and elevation maps for every pixel. Sign reversals in the gradient maps correspond to traversals across visual areas, which help locate visual areas (Sereno et al., 1994). Based on these maps, we chose a target region for parietal cortex to overlap with area A/RL and to be adjacent to primary visual cortex (V1). This region is defined as a parietal area in the Allen Mouse Brain Common Coordinate Framework (Wang et al., 2020).

Two-photon imaging

Two-photon imaging was performed in the target location using a Thorlabs B-Scope with a Nikon 16x 0.8 NA water immersion objective. A Ti:Sapphire laser (Chameleon Ultra II, Coherent Inc.) provided excitation at 920 nm, with depth-adjusted power level controlled by an electro-optic modulator (Pockels cell, M350-80LA, Conoptics Inc.). A custom metal cylinder, cone and black cloth prevented light contamination from the illuminated screens. Acquisition was controlled using ScanImage (Pologruto et al., 2003), and frames were acquired continuously at 30 Hz over an imaging window of 500x500 μm, at a resolution of 512x512 pixels. Multi-plane imaging was performed using a piezo motor over two planes in layer 2/3, starting at 90-130 μm below the surface, separated by 60-70 μm, spanning a total of 180-210 μm. The effective imaging rate was 10 Hz per plane (the fly-back plane was discarded).

At the beginning of each acquisition, the mean image over several frames of the previous task's recording plane was used as a reference plane, and the live movie of the current imaging plane was manually aligned in z, x, and y, to match until the difference was indistinguishable to the eye. Following acquisition, the raw movies were then examined by eye to ensure that the same population of cells was visible. Imaging sessions were dropped if a large proportion of neurons were no longer visible by the end of each task or across both tasks. This realignment procedure was most important when switching between the tasks, and comparing activity across recording days. This procedure was not needed between steering wheel and hybrid task acquisitions as the mouse remained in the same head fixation position to perform these tasks.

The movies comprising all conditions within a given imaging session (the two tasks plus other conditions) were concatenated before processing in Suite2p (Pachitariu et al., 2017) for motion correction (registration), cell detection, signal extraction, neuropil correction and spike deconvolution. Neuropil was estimated as a radius of size 5x the number of pixels defined for the cell and subtracted from cell activity using a multiplicative coefficient estimated per cell, usually ~0.6-0.8. Deconvolution was performed using the OASIS algorithm (Friedrich et al., 2017) wrapper within Suite2p. Regions of interest (ROIs) detected by Suite2p were manually curated using the Suite2p Graphical User Interface. ROIs were classified as cells according to spatial and temporal criteria, i.e., that the ROI reasonably resembled a disc-like soma at the size expected at the imaging zoom used and that the inspected activity trace had good signal-to-noise. Manual curation was performed blind to the time at which the task transition occurred.

To judge consistency of the results, we returned to the same cells across days, using RegisterS2p to align recorded ROIs across days and identify matches (Pachitariu et al., 2017). We only analyzed neighboring pairs of days (separated by one or two days) as this ensured that recorded cells were most similar, with respect to morphology, cell death and changing neural representations across longer timescales if any. Pairs of sessions were upheld to the same strict criteria for inclusion as described above, so n = 4 pairs of days remained for analysis. The cells analyzed were the union of cells present in each pair, and we analyzed pairs of task conditions, either the same task across days, or different tasks across days.

Data analysis

All analyses were carried out on a session-by-session basis. Summary statistics were then taken across sessions.

Out-of-focus fluorescence from neuropil (dendrites and passing axons) can erroneously contribute to the signal averaged within the pixels that define a cell. A standard procedure to correct for this neuropil is to subtract a scalar multiple of the average activity in a radius around each cell (e.g., Chen et al., 2013b; Dipoppa et al., 2018). We estimated this scalar ("neuropil coefficient") for each cell and found it to be usually ~0.6-0.8. Meanwhile, standard cell extraction procedures for two-photon data involve estimating pixels which are correlated within themselves but not with respect to the surrounding pixels in the background, i.e., the neuropil. Given these well-established assumptions about what constitutes a cell and what constitutes noise to be subtracted out, it is reasonable to take the neuropil as an estimate of baseline noise. Indeed, deviation from neuropil has been used as a metric for selecting active neurons (Chen et al., 2013a; Chen et al., 2015).

To summarize each cell's activity across the whole session we thus used "isolation distance", which captures deviations of a cell's activity relative to its neuropil surround (Stringer and Pachitariu, 2019), akin to a signal-to-noise ratio. Specifically, for each cell and its respective neuropil surround (both estimated in Suite2p) the matrix of pixels x time is concatenated over pixels, and the mean (over both cell and neuropil) is subtracted over time. Then singular value decomposition is used to reduce the dimensionality to the first principal component, resulting in a one-dimensional summary per pixel. To measure the distance between the distributions of the pixels corresponding to the cell and neuropil, we used the Bhattacharyya distance, which accounts for the variance of each distribution, since the neuropil distribution tends to have less variance (over the range spanned by the cell, which has peaks of fluorescence that correspond to activity).

Isolation distance produced results qualitatively similar to common measures (mean, standard deviation, skewness, coefficient of variation) but was most robust to baseline noise. This last requirement was especially important as most of our mice (5/6) came from a GCaMP6s transgenic strain that has a higher baseline noise floor (Huang et al., 2021).

Encoding models

To assess task-relevant encoding, we characterized each neuron's deconvolved calcium activity with a weighted combination of task events using ridge regression, a common method applied to two-photon calcium imaging data (e.g., Diamanti et al., 2021; Ramesh et al., 2018) and electrophysiology data (e.g., Steinmetz et al., 2019). Specifically, we fit single neuron activity using behavioral predictors, both temporally defined events (stimulus onset, choice, reward) and continuous events (velocity of the task apparatus). We used a window around each event to capture both predictive and consequent activity related to each event (for stimulus onset: -50 to 500 ms; choice: -150 to 400 ms; reward: -100 to 400 ms; for continuous variables: -500 to 500 ms with steps of 250 ms). We used both

'linear' and 'glm' fits with similar results. We performed ridge regression, which regularizes the regression coefficients to have small values with a parameter λ that was set through 11-fold cross validation (out of a range [0.01 0.05 0.1 0.5 1]). To measure the extent of task-relevant encoding we used percentage variance explained.

For the steering-wheel task, we used the following predictors: stimulus onset (left or right side), choice (left or right side), reward, and steering wheel turns (counter-clockwise or clockwise). For the T-maze, we used three models. One model resembled the steering-wheel model and had the following predictors: stimulus onset (left or right), choice (left or right), reward, forward velocity, and turning (counterclockwise or clockwise). Another model resembled previous models applied to parietal activity in the T-maze (Krumin et al., 2018) and had the following predictors: position down the corridor (z), heading angle (theta), reward, forward velocity and turning (counterclockwise or clockwise). Finally, we also directly applied the T-maze position-heading model used in Krumin et al. (2018). With regards to the main results, these three models performed similarly.

Running and stationarity

To determine if task selectivity could be explained by running modulation, we assessed a neuron's running modulation in the "passive ball" condition. Running modulation was computed as the correlation between each cell's activity and the mouse's running speed. The deconvolved, neuropil-corrected calcium trace was used to account for movement artefacts which can occur due to fast z-drift (Stringer, 2018). The running speed was taken as forward movement on the ball, and was downsampled to match the imaging frame rate. Both the running speed and neural activity were smoothed by convolving the traces with a 1-s s.d. Gaussian filter. A permutation test was used to assess significance by circularly shifting running speed relative to the neural activity 1,000 times by a random number of frames.

Other movement analyses

On most sessions, we concurrently recorded video of the mouse's face, either zoomed in to the eye, or the whole face (including snout and whiskers). After acquisition, videos were checked to ensure clear video resolution. Each acquisition was then individually processed to estimate either pupil diameter (using an in-house eye tracking software) or face and whisker motion using FaceMap (Stringer et al., 2019). Whisker motion was estimated by manually selecting a box around the whisker pad, and taking the absolute motion energy, while facial motion was estimated by cropping around the side-view of the mouse's snout, and taking the first principal component (Stringer et al., 2019). The resulting sessions with good movement data were n = 2 for pupil diameter and n = 13 for face/whisker motion. Modulation by movement variables (using a Pearson correlation) was performed separately for each task condition, and a permutation test was used to assess significance by circularly shifting the movement trace relative to the neural activity 1,000 times by a random number of frames, for each neuron. Where possible, we compared sessions across days, both within and across tasks.

Choice selectivity

To determine choice selectivity, we used the mean deconvolved calcium activity over the whole trial, from stimulus presentation and including the motor execution of the choice. In some sessions, the same stimulus condition was repeated if the mouse did not respond correctly, to encourage engagement. These repeated trials were excluded from analyses, as mice could know with certainty the correct choice even prior to the trial, and thus may engage in a different strategy for choices that is not guided by sensory evidence. Trials in which the wheel moved <125 ms after stimulus onset were discarded as such movements are unlikely to be a response to the stimulus. Sessions were only included if at least 10 trials of each comparison (e.g., 10 left-side choices and 10 right-side choices) remained after excluding these invalid trials defined above. For analyses comparing choice selectivity across tasks, there needed to be 10 trials for each choice and each task for a session to be included.

In well-performing mice, stimulus and choice are highly correlated; to disentangle these factors and focus on choice alone, we used "combined conditions choice probability" (ccCP, Steinmetz et al., 2019). Choice probability is classically calculated as the area under a receiver operating characteristic (ROC) curve i.e., the probability that firing rate in the trials with one choice is greater than in the trials with the other choice (Britten et al., 1996). ROC is equivalent to a Mann–Whitney *U* statistic, so can be calculated by comparing each trial of one condition (i.e., contrast value and side) to each of the other conditions, counting the number of such comparisons for which the first condition wins, and dividing by the total number of comparisons. To extend the method to a situation of many stimulus conditions but few trials of each condition, we add the numerators and denominators of this ratio across the full stimulus set (nine contrast conditions), and then divide. We normalized the ccCP to lie between -1 and 1, where negative values mean higher activity during left choices. To assess the significance of these values we used a permutation test: for every neuron, trial labels were shuffled 1,000 times, and choice selectivity was recomputed for each new batch of pseudo "left"-labeled and "right"-labeled trials. As the same number of trials remain in each comparison, this test accounts for imbalanced samples of each condition. To compare choice selectivity across tasks, we only used the subset of neurons active in both tasks, chosen by a threshold of isolation distance > 0.3. Sessions with <10 neurons active in both tasks were excluded from this analysis.

Neuron, Volume *110*

Supplemental information

Task specificity

in mouse parietal cortex

Julie J. Lee, Michael Krumin, Kenneth D. Harris, and Matteo Carandini

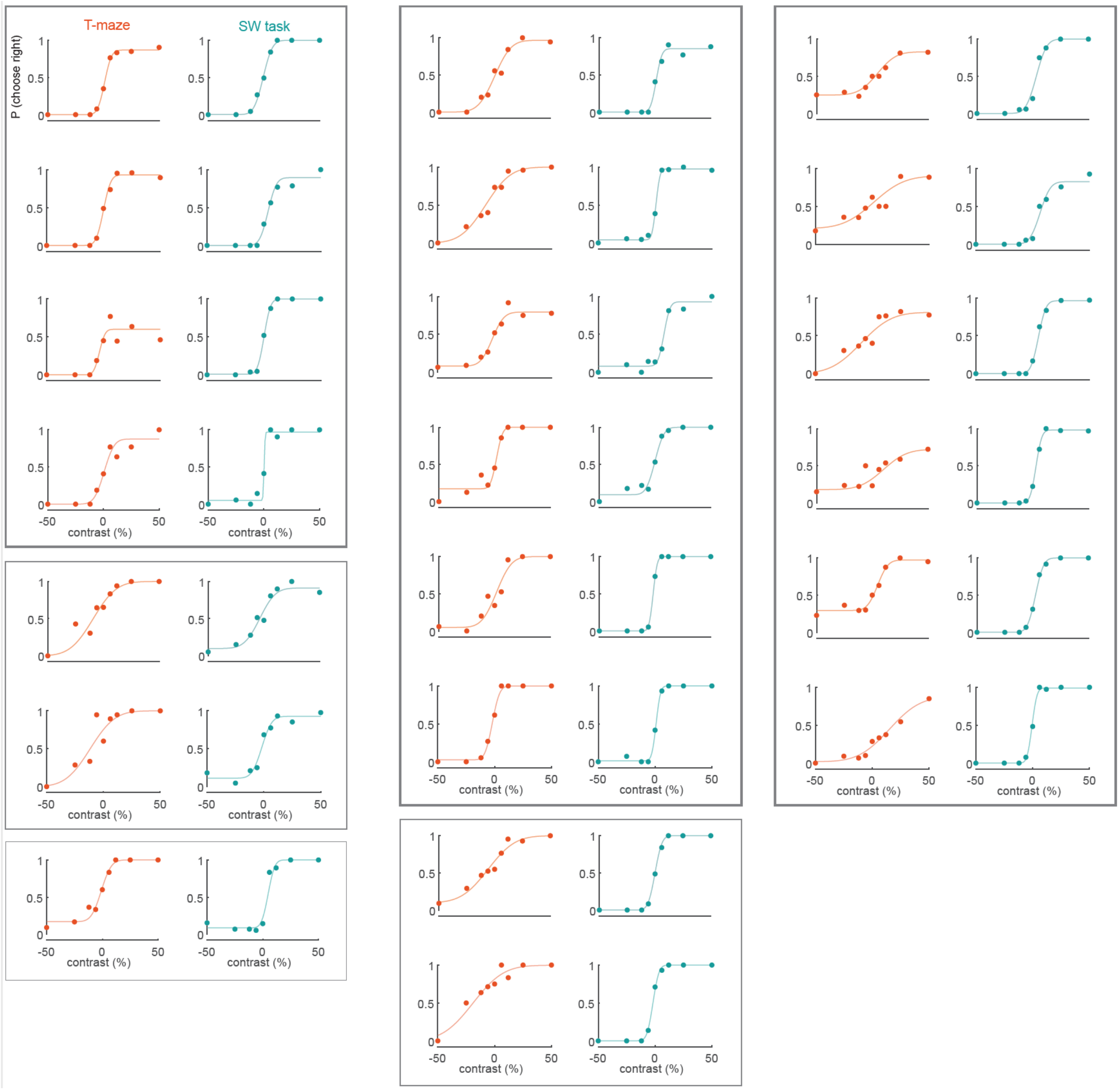

Supplementary Figure S1. Psychometric data for each task, session, and mouse. Related to Figure 1.
For each of six trained mice (boxes), we measured neural activity in one or more sessions (rows). Each session included two tasks performed consecutively: T-maze (*orange*, left) and Steering Wheel task (*cyan*, right). Tasks were not necessarily presented in the order shown.

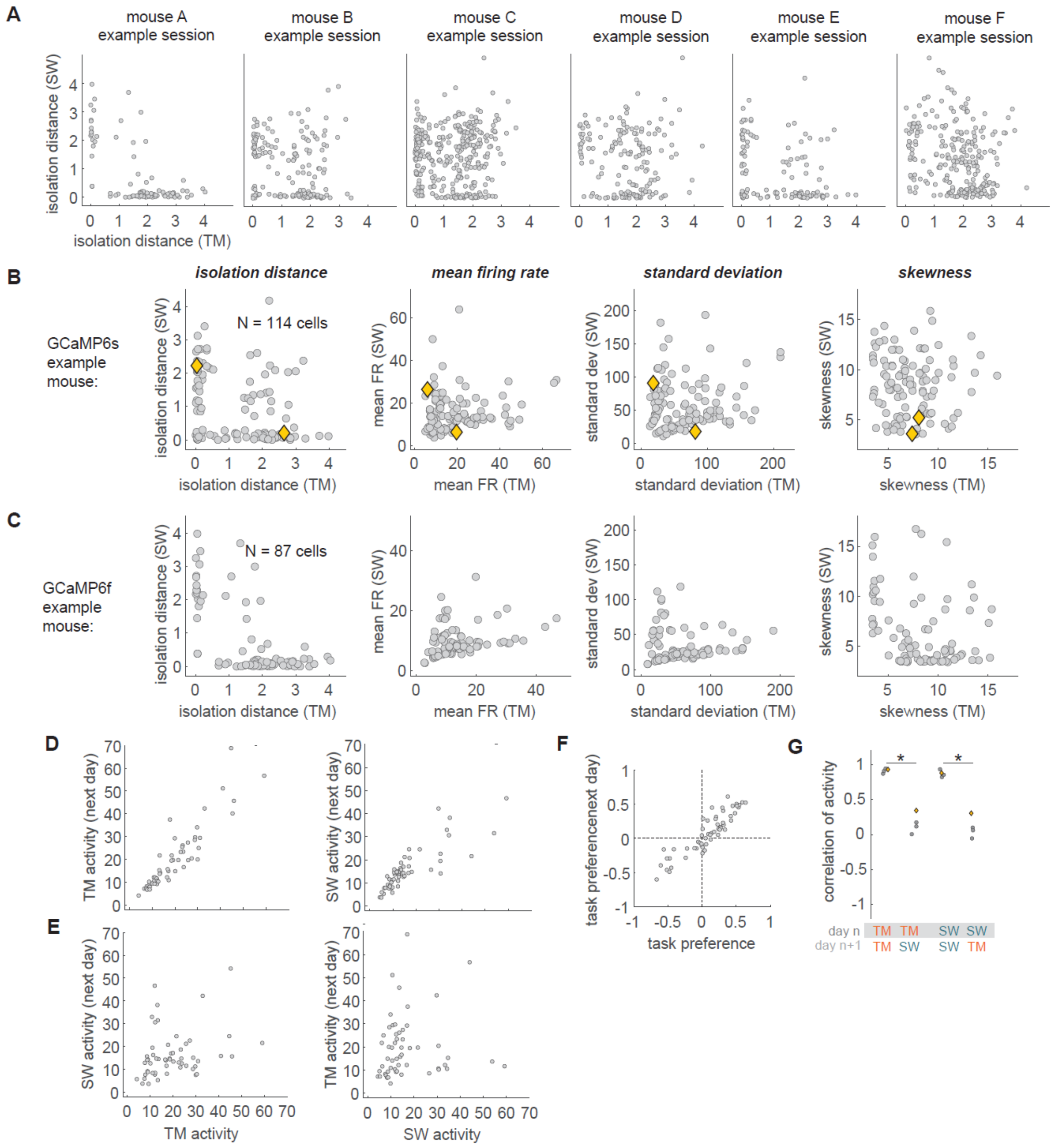

Supplementary Figure S2. Further examples and alternative measures of activity. Related to Figure 1.
(A) Further example sessions (one per mouse) showing activity (isolation distance) across tasks (as in Figure 1E).
(B) Comparison of activity in the T-maze (TM, abscissa) vs. steering wheel task (SW, ordinate) for the example session in Fig. 1E (in a mouse expressing GCaMP6s, mouse E), using different measures for calculating activity: isolation distance, mean, standard deviation and skewness. *Diamonds* indicate the same neurons in all plots.
(C) Same as B, for an example session from a GCaMP6f mouse (mouse A).
(D-G) Reproduction of analyses from Figs 2B-E using mean firing rate as the measure of activity instead of isolation distance. Mean firing rate was more similar across than within tasks.

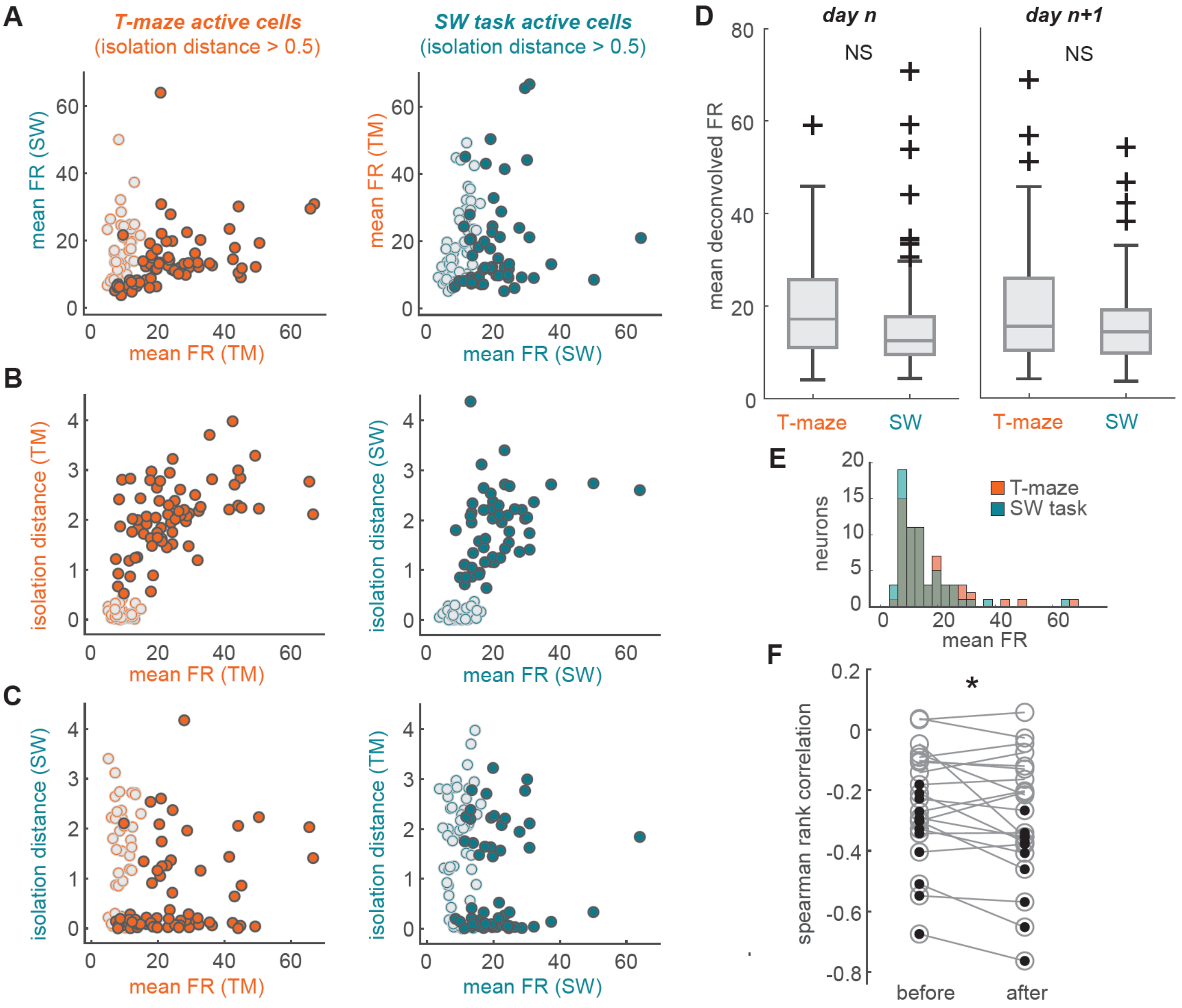

Supplementary Figure S3. Effects of mean firing rate on isolation distance and correlations. Related to Figure 1.

(A) Comparison of mean firing rate across tasks in an example session for cells with some activity (isolation distance > 0.5, *filled circles*) in the T-maze task (*left)* or the steering-wheel task (*right*).

(B) Same format, comparing mean firing rate and isolation distance within the same task. There is a positive relationship between the two measures.

(C) Same format, comparing mean firing rate and isolation distance across tasks. A negative relationship means high firing rate in one task predicts low activity in the other task (i.e., task selectivity); a positive relationship means high firing rate in one task predicts high activity in the other task (i.e., task generality). Here, the T-maze (*bottom left*) had a slight significant correlation, r = 0.25, p = 0.04, and the steering wheel task (*bottom right*) did not have a significant correlation, p = 0.65. However, across sessions, there was a small but significant positive relationship using a linear mixed-effects model: other task's isolation distance ~ mean FR + (mean FR | session), p = 0.02. That is, neurons with higher firing rate were slightly more likely to be active in both tasks.

(D) Mean firing rate (FR) across tasks, for the example session in Fig. 2. Here, mean FRs in the T-maze and SW task were not significantly different (rank-sum test). However, in other sessions (13/21), firing rate was higher in the T-maze. Indeed, task was a significant predictor of mean FR (p < 0.01, linear mixed-effect model with session as a random effect, FR ~ task + (task | session)).

(E) To test for the effect of firing rate, we subsampled neurons so that their mean firing distributions for each task (orange and blue histograms) were statistically indistinguishable (p ⩾ 0.05 in a rank-sum test). This histogram shows results for an example session.

(F) Subsampling to equate firing rate distributions often made the Spearman rank correlation of isolation distance more negative across tasks (filled circles denote sessions where p < 0.05 comparing before and after subsampling). This tendency was significant across sessions (p = 0.04, one-tailed sign-rank test). Therefore, differences in mean firing rate across tasks do not account for our findings, as similar firing rates produce the same results.

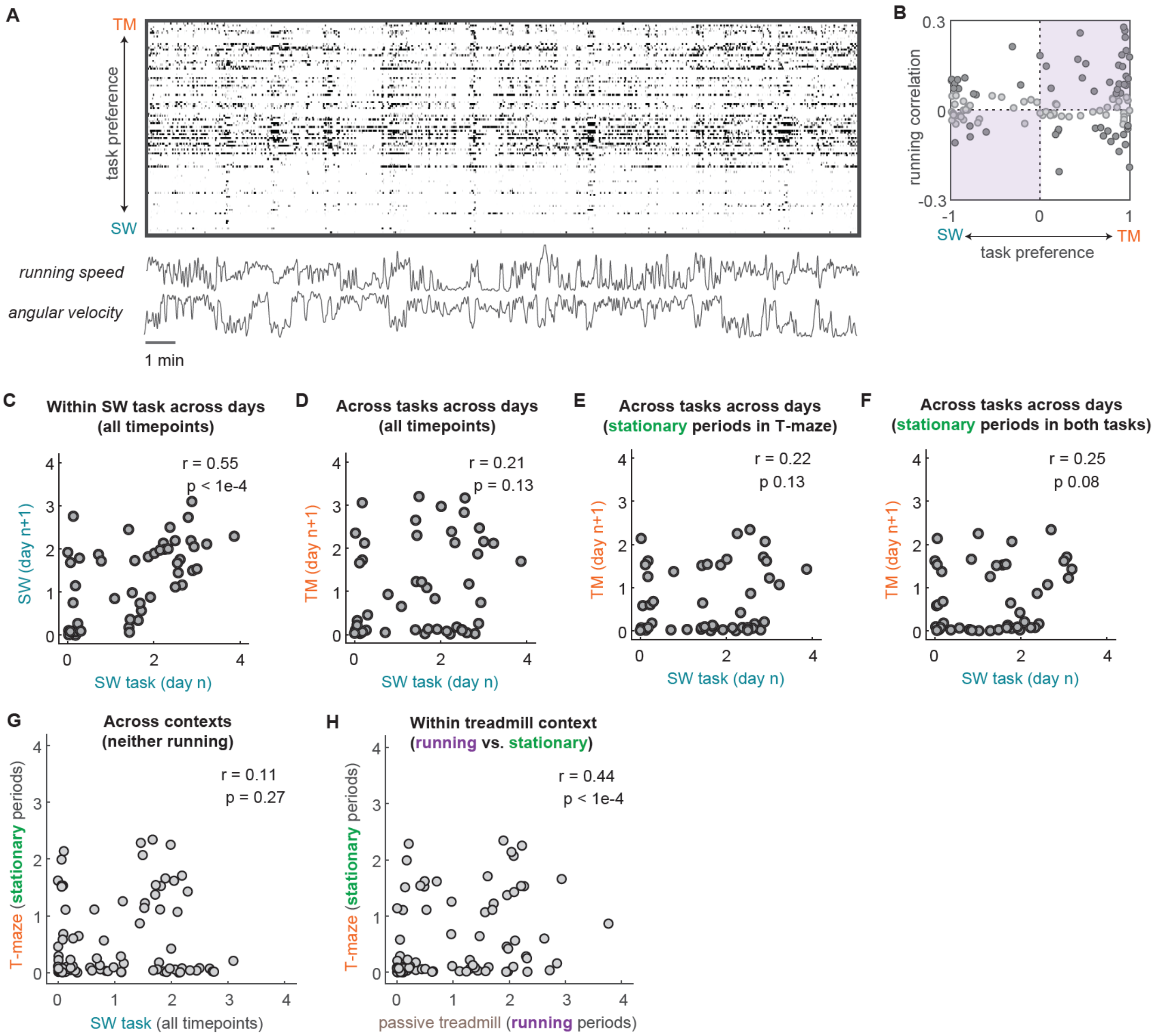

Supplementary Figure S4. Running does not explain task specificity. Related to Figure 3.
(A) Firing rate of neurons, sorted by task selectivity, in an example session during the passive treadmill condition. Below: running speed and angular velocity on the ball.
(B) Running modulation does not explain task preference. Positive vs. negative values on the y-axis indicate preference for T-maze vs. steering wheel (SW) task. *Filled circles*: neurons with significant running modulation (permutation test). *Purple overlay*: hypothetical distributions of cells, if T-maze neurons were just those modulated by running, and SW task neurons were just those suppressed by running. In this session, the Spearman rank correlation was not significant, using all neurons, r = 0.04, p = 0.67, or just significantly running-modulated neurons (filled circles), r = 0.06, p = 0.65.
(C) Comparing steering-wheel (SW) task activity across two days; activity is highly correlated (r = 0.55, p < 1e-4).
(D) Same example days, comparing across tasks; activity is not significantly correlated (r = 0.21, p = 0.13).
(E) Same as D, measuring T-maze activity only during stationary periods (running speed < 1.2 cm/s); activity is not significantly correlated (r = 0.22, p = 0.13).
(F) Same as D, using only stationary periods in both tasks; activity is not significantly correlated (r = 0.25, p = 0.08).
(G) Another session where activity in the T-maze task during stationarity is not correlated with activity in the steering-wheel task (r = 0.11, p = 0.27). This mirrors the results of activity from Fig 1E. The same result was observed in another session (not shown, r = 0.04 p = 0.73). This result holds even when we additionally excluded periods of steering wheel movements, i.e., forelimb stationarity (this session: r = 0.18, p = 0.08; another: r = -0.01, p = 0.95). In summary, activity was not significantly correlated across contexts, even within the same movement condition (stationarity).
(H) Same session as in (G). Activity in the T-maze task during stationarity is correlated with the passive treadmill condition during running (r = 0.44, p < 1e-4). The same pattern was seen in another session (not shown, r = 0.37, p < 0.01). This extends the results of correlated activity between task and passive conditions within the T-maze context (Fig 2B), and shows that this correlation holds even across movement conditions (running vs stationary).

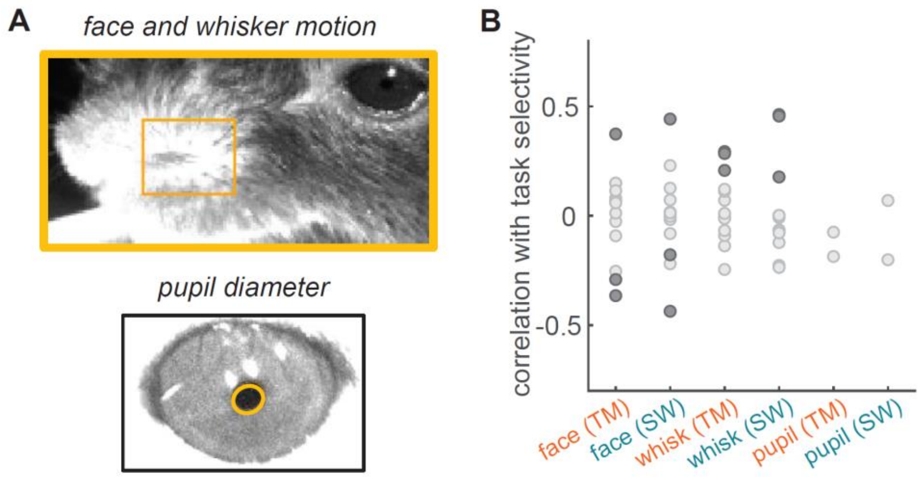

Supplementary Figure S5. Facial movements and pupil diameter do not explain task specificity. Related to Figure 3.
(A) Video frame from sessions with high-resolution video of face (*top*) or eye (*bottom*). Face motion was extracted from the whole face image (large yellow rectangle), whisker motion from a smaller region (smaller rectangle), and pupil diameter from a fitted ellipse (yellow contour). Facial movements, whisker motion and pupil diameter were estimated separately in each task and session (Methods).
(B) Summary of correlations of movement modulation with task selectivity, as in Supplementary Fig. S4B, although estimated within each task condition. Each point is a session. Filled circles are significant correlations ($p < 0.05$, 3/13 sessions for facial movements and whisking, 0/2 sessions for pupil diameter).

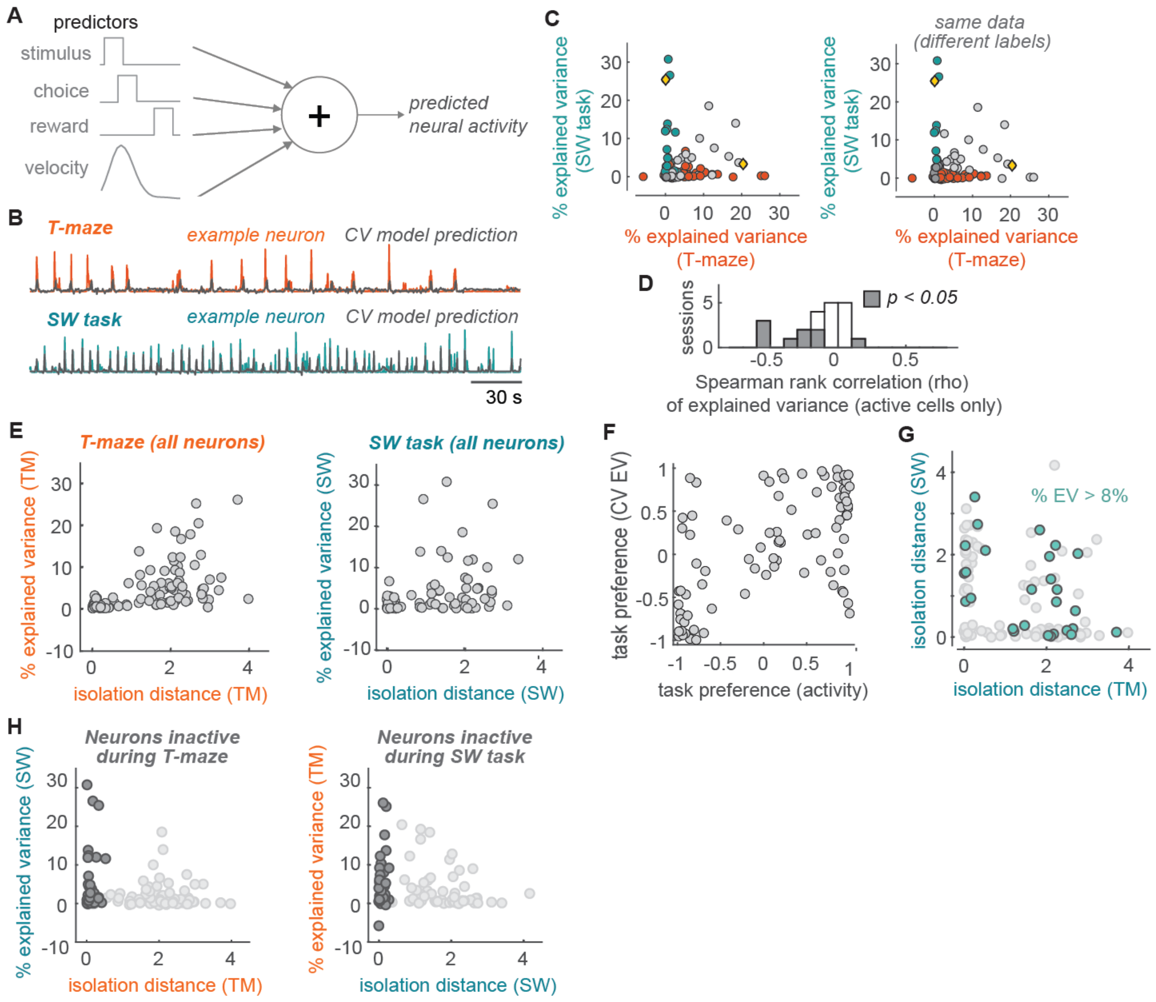

Supplementary Figure S6. Encoding of task variables is task specific. Related to Figure 4.

(A) Schematic of an encoding model based on behavioral predictors: stimulus (left vs right, or zero contrast), choice (left vs right), reward, and velocity of the respective apparatus. The model was fit separately to each task.

(B) Magnified traces of two neurons: one that responded in the T-maze task (*top*) and one that responded in the Steering Wheel task (*bottom*), showing deconvolved calcium activity (*color*) and model predictions (*gray*).

(C) Cross-validated explained variance (%) by each encoding model for the two tasks, in the example session of Fig. 1E. Example neurons in (B) are indicated by diamonds. Spearman rank correlation across tasks: r = 0.08, p = 0.38. We fitted a two-dimensional Gaussian mixture model to this data. The model returns cluster labels by determining the probability of cluster assignment according to a prespecified number of clusters. This method is the same as used to label the clusters in existing Figure 1E. *Left:* Neurons (dots) are colored according to Fig. 1E. *Right:* neurons are fitted according to % explained variance.

(D) Summary over sessions showing Spearman rank correlation of cross-validated explained variance across tasks, for neurons active in either or both tasks (isolation distance > 0.5). *Filled* bars indicate significance (9/21 sessions).

(E) Relationship between activity (isolation distance) and encoding of task variables (% cross-validated variance explained) for the T-maze (*left*) and the SW task (*right*), for all neurons in the example session from Figs 2 and 5.

(F) Same data as in (A) comparing task preference measured by activity vs task preference measured by cross-validated variance explained by the encoding models. The two measures were well-correlated, r = 0.62, p < 0.001.

(G) Same data as in (A) comparing isolation distance across tasks for the subset of neurons encoding task-relevant information in either task (*cyan*: >8% cross-validated explained variance in the T-maze, steering-wheel task, or both). Spearman rank correlation, r = -0.48, p = 0.003. Across all sessions, 15/21 sessions had significant negative correlations (p<0.05) and 6/21 were not significantly correlated; median r = -0.47.

(H) Same data as in (A), comparing activity and encoding across tasks. Filled circles indicate neurons inactive in one task (isolation distance < 0.5; silent neurons were excluded). These neurons tend to encode task-relevant activity in the other task. Explained variance by task-related events was significantly (p < 0.05) greater than 0 in 12/21 sessions for task encoding in the SW task for neurons inactive in the T-maze (e.g., *left*) and for task encoding in the T-maze in 20/21 sessions for neurons inactive in the SW task (e.g., *right*). Significance of task encoding was assessed according to a permutation test where the median cross-validated explained variance (of inactive cells in the other task) was compared to permutations where the labels of inactive vs active cells were shuffled randomly 1000 times and the median explained variance of these pseudo-inactive populations was computed. The explained variance was considered significant if the actual median was greater than the 95th percentile (alpha level = 0.05) of the permutations.

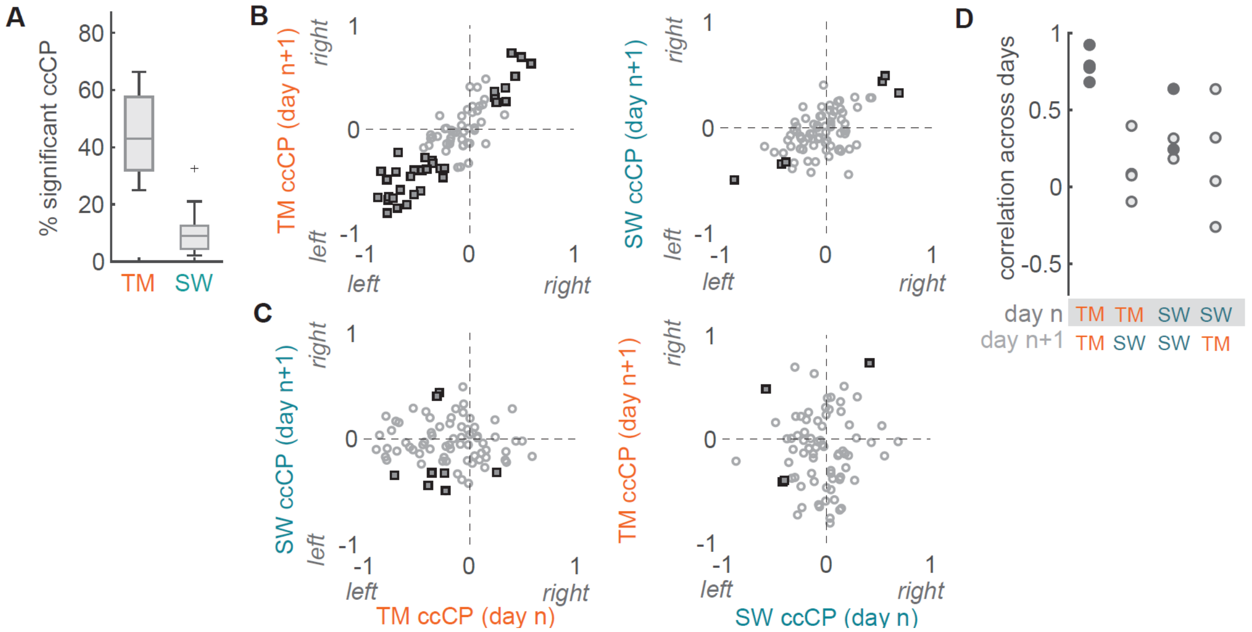

Supplementary Figure S7. Preference for ongoing choice is not shared across tasks. Related to Figure 4.

(A) Proportion of neurons with significant choice selectivity (ccCP) over 21 sessions from 6 mice, for neurons with at least a little activity (isolation distance > 0.3) in each task (both task-selective and task-general neurons). Outliers are denoted by a *plus* symbol. Across sessions, there was no significant difference between the number of choice-selective neurons in the task-selective and task-general populations (isolation distance > 0.5 in only one vs. both tasks), for either the T-maze ($p = 0.84$, paired sign rank test) or SW task ($p = 0.67$). For the T-maze, median number of choice-selective neurons in the task-selective population: 28; in the task-general population: 9.5. For the SW task, median number of choice-selective neurons in the task-selective population: 4.0; in the task-general population: 4.5.

(B-C) Choice selectivity (ccCP) across days within tasks (B) and across tasks (C) for neurons showing some activity in both tasks (isolation distance > 0.3 in both tasks). Negative values denote preferences for left choices, positive for right choices. *Filled squares* denote neurons that were significantly selective for choice in either day ($p<0.05$). Out of the larger recorded population, the proportion of neurons active in both tasks plotted in (C) was 23.4% for one cross-task comparison (*left*) and 26.0% for the other (*right*).

(D) Correlation of ccCP across days for different combinations of tasks, for n = 3 mice, four pairs of days). Neurons were selected as in C-F (isolation distance > 0.3 in both tasks. Spearman rank correlation (f*illed circles*) was only significant within tasks (across days), not across tasks.
